# Use of hydrophilic pharmaceutical excipients to modulate release of metal ions from silicone elastomers

**DOI:** 10.1080/10717544.2025.2545515

**Published:** 2025-08-21

**Authors:** Xin Shen, Xinyu Zhao, Clare F. McCoy, Yahya H. Dallal Bashi, Peter Boyd, R. Karl Malcolm

**Affiliations:** a School of Pharmacy, Queen’s University Belfast, Belfast, UK; b College of Pharmacy, University of Sharjah, Sharjah, United Arab Emirates

**Keywords:** Drug delivery, gelatin, polyvinylpyrrolidone, hydroxypropyl methylcellulose, sucrose, silicone rubber, vaginal rings

## Abstract

Silicone elastomers based on polydimethylsiloxane are biocompatible and non-biodegradable thermosetting polymers used in various drug delivery applications, including subdermal implants, vaginal rings, and intrauterine devices. Without exception, all marketed silicone elastomer drug delivery products provide sustained or controlled release of highly hydrophobic small drug molecules, since drug solubility in the silicone matrix is a prerequisite for molecular diffusion and release. We are interested in developing multipurpose silicone elastomer vaginal rings for local administration of metal ions—such as copper and zinc—for non-hormonal contraception and antimicrobial therapy. However, sustained/controlled release of metal ions from silicone elastomers containing metal nanopowders or metal salts is challenging due to their limited solubility in silicone. In this study, we assess the potential for enhancing the release of copper or zinc ions from silicone elastomer devices by co-formulating copper nanopowder, zinc nanopowder, copper sulfate, or zinc acetate with four common pharmaceutical excipients—gelatin, polyvinylpyrrolidone, sucrose, and hydroxypropyl methylcellulose. The study demonstrates that (i) copper/zinc nanopowders and salts can be successfully incorporated into addition-cure silicone elastomers, (ii) *in vitro* release of Cu^2+^/Zn^2^ ions from silicone elastomers loaded with divalent salts was ∼100 times greater compared to nanopowders; (iii) incorporation of gelatin, polyvinylpyrrolidone, sucrose and hydroxypropyl methylcellulose significantly enhanced the release of Cu^2+^/Zn^2+^ ions (up to ∼30-fold), and (iv) enhanced release was due to water absorption into the silicone elastomer devices, causing swelling of the devices to an extent proportional to the excipient loading.

## 1. Introduction

Silicone elastomers are used widely in drug delivery devices due to their biocompatibility, chemical stability, and ability to control the rate of drug release (Mashak et al. 2009; Aliyar and Schalau 2015). For example, various marketed vaginal ring products are fabricated from silicone elastomers (Estring^®^, Femring^®^, Progering^®^, Annovera^®^, and DapiRing^®^) (Boyd et al. 2019; Murphy et al. 2019); Jadelle^®^ and Sini-implant (II)^®^ (Levoplant^®^) are reservoir-type silicone elastomer subdermal implants for long-term administration of the contraceptive progestin levonorgestrel (Benagiano et al. 2008); and many contraceptive intrauterine devices (e.g. Mirena^®^, Kyleena^®^, and Skyla^®^) comprise a silicone elastomer levonorgetrel-loaded reservoir and a non-medicated silicone elastomer rate-controlling membrane to control release (Bao et al. 2018). Irrespective of the design of these marketed devices, drug release is invariably achieved bymolecular diffusion of the dissolved drug molecules in the silicone elastomer matrix (Mashak et al. 2009). Therefore, drug release rates from silicone elastomer devices are dependent upon factors such as drug solubility in the silicone elastomer, drug diffusivity in the silicone elastomer, and device surface area (Roseman and Higuchi 1970; Malcolm et al. 2003). Commercial silicone elastomer materials commonly used for drug delivery applications are based on polydimethylsiloxane, which is highly hydrophobic due to the methyl groups orientating toward the surface and creating a low surface energy. Therefore, it is not surprising that drug delivery products constructed from silicone elastomer are almost exclusively used for administration of highly hydrophobic and highly potent drugs (Ghannam et al. 1986; McBride, Boyd, et al. 2019).

Various strategies have been reported for enhancing drug release from silicone elastomers and extending release to more hydrophilic drugs (Table 1) (Hsieh et al. 1985; Carelli et al. 1989; Li and Peck 1989; Lopour et al. 1990; Dahl and Sue 1992; Di Colo 1992; Hu et al. 2000; Kajihara et al. 2000, 2001, 2003; Maeda, Brandon, et al. 2003; Maeda, Ohashi, et al. 2003; Woolfson et al. 2003; Manosroi et al. 2005; Woolfson et al. 2006; Brook et al. 2008; Mashak 2008; McBride et al. 2009; Rajendra et al. 2010; Morrow et al. 2011; Snorradóttir et al. 2011; McConville et al. 2012; Tolia and Li 2012; Forbes et al. 2014; Nemati et al. 2014; Murphy et al. 2016; Ma et al. 2018; Mazurek et al. 2018; Farahmandghavi et al. 2019; McBride, Boyd, et al. 2019; Mikolaszek et al. 2020; Mazurek, Frederiksen, et al. 2021; Mazurek, Yuusuf, et al. 2021; Fanse et al. 2024). Most commonly, these involve incorporation of hydrophilic excipients into the silicone elastomer matrix. Upon implantation or during *in vitro* release testing, fluid is absorbed into the silicone matrix leading to enhanced drug solubility. When sufficiently high concentrations of excipients are used, aqueous pores and channels are formed in the silicone matrix through which solvated drug molecules can more easily diffuse (Di Colo et al. 1983, 1986; Carelli et al. 1987, 1988, 1989, 1995).

**Table 1.** Reported strategies for modulation of drug release from silicone elastomers.

Strategy	Mechanism	Description	References
Incorporation of hydrophilic excipients	Introduce pores to create water channels	HPMC, sucrose, or glycine dispersed in silicone to increase bovine serum albumin (BSA) release	Carelli et al. 1989; Woolfson et al. 2006; Brook et al. 2008; Di Colo 1992; Morrow et al. 2011; Snorradóttir et al. 2011; Murphy et al. 2016; Mazurek et al. 2018; McBride, Boyd, et al. 2019; Mazurek, Frederiksen, et al. 2021; Mazurek, Yuusuf, et al. 2021
Hydrophilic polymer blending	Blend silicone with polymers to enhance water uptake and drug mobility	Silicone–polyethylene glycol (PEG) copolymer matrices used to enhance the permeability of the silicone elastomer to hydrophilic and ionic species	Li and Peck 1989; Rajendra et al. 2010; Forbes et al. 2014; Mikolaszek et al. 2020
Porous matrix	Introduction of pores to promote diffusion	Silicone containing PEG8000-leached pores to enhance acetaminophen and tartrazine release	Dahl and Sue 1992; Nemati et al. 2014
Use of co-solvents or solubilizers	Enhancing drug mobility by reducing matrix–drug interactions and lowering diffusion activation energy	Glycerol and NaCl incorporated into silicone elastomer implants to enhance release of melatonin and estradiol	Hsieh et al. 1985; Maeda, Brandon, et al. 2003
Incorporation of preformed hydrogel particles	Embedding hydrophilic hydrogel particles that swell upon hydration to form aqueous diffusion paths	Polyacrylamide hydrogel particles incorporated in silicone rubber to enhance progesterone release	Lopour et al. 1990; Mashak 2008
Drug molecular complexation	Modify drug solubility and partitioning *via* inclusion complexation	Azelaic acid-hydroxypropyl-β-cyclodextrin complex used in silicone membrane to enhance the release of azelaic acid	Manosroi et al. 2005
Surface hydrophilization	Graft hydrophilic groups on silicone surface	PEGylation of silicone elastomer to modulate triclosan release	McBride et al. 2009
Drug particle size engineering	Altering particle size to modify surface area and diffusion dynamics	Larger particle size of interferon/human serum albumin (HSA) powder in silicone elastomer to increase release rate of IFN	Kajihara et al. 2000; Farahmandghavi et al. 2019
Polymer microstructure engineering	Using additives/fillers to modify polymer crystallinity, viscosity, drug affinity, and diffusion paths	Silica, PEG, or silicone oil used to modify polydimethylsiloxanes for optimized levonorgestrel release	McConville et al. 2012; Tolia and Li 2012; Ma et al. 2018; Fanse et al. 2024
Formulation geometry design	Control of release via physical structure to maintain a constant diffusion area	A 1/8 fractional segment core design of vaginal ring to achieve zero-order release of oxybutynin	Kajihara et al. 2001, 2003; Maeda, Ohashi, et al. 2003; Woolfson et al. 2003
Stimuli-responsive additives	Triggering release via stimuli, such as pH, enzymes, or temperature	N-isopropylacrylamide hydrogel particles incorporated into a silicone rubber membrane as temperature-responsive additives that enhance release at temperature > 34 °C	Hu et al. 2000; Nemati et al. 2014

Metal ions and inorganic salts are used as active pharmaceutical ingredients: magnesium and aluminum salts as antacids; iron, zinc, calcium, and magnesium supplements to treat deficiencies; and copper ions for intrauterine contraception. We are interested in developing silicone elastomer vaginal rings releasing copper or zinc ions locally to the human vagina for the purpose of non-hormonal contraception (Shen et al. 2025). Mimicking the well-established mechanism of copper intrauterine devices (IUDs), divalent copper and zinc ions can adversely impact sperm motility by disrupting the function of sperm cell membranes and enzymes, such that the sperm struggle to penetrate cervical mucus (Jecht and Bernstein 1973; Holland and White 1988; Roblero et al. 1996; Huang et al. 2001). Specifically, these ions generate reactive oxygen species (ROS) which compromise membrane integrity, impairing sperm movement. They also interfere with enzymes involved in sperm metabolism and motility, such as those related to ATP production, reducing energy availability for movement. Together, these effects reduce sperm viability and motility, contributing to contraceptive efficacy. We are not seeking or anticipating systemic uptake of the copper/zinc ions; given the charge on the ions and their ability to complex with proteins or phosphate ions in the vagina, their passive diffusion across epithelial membranes will be limited. For example, copper IUDs release Cu^2+^ ions locally into the uterine cavity, but show minimal systemic absorption; instead, the copper acts locally, exerting a spermicidal effect and inducing an inflammatory response in the endometrium.

However, achieving sustained or controlled release of metal salts/ions from silicone elastomers is challenging given the poor solubility of metal ions in silicone. One strategy to overcome the poor solubility is to load the device with relatively large initial concentrations of the metal/metal salt. For example, incorporation of up to 25% w/w copper nanoparticles into silicone elastomer matrices produced concentration-dependent and near steady state release of copper ions after an initial burst (Xu et al. 2013). Yet, inclusion of such high active loadings in a drug product can be prohibitively expensive; for context, most marketed drug-releasing vaginal rings contain significantly <1% w/w active, e.g. NuvaRing^®^ (0.54%), Estring^®^ (0.02%), and DapiRing^®^ (0.31%). An alternative and less expensive strategy would be to include a hydrophilic pharmaceutical excipient into the ring device alongside a much smaller loading of the active. In this study, four pharmaceutical excipients—gelatin, polyvinylpyrrolidone (PVP), hydroxypropyl methylcellulose (HPMC) and sucrose—were selected (Table 2) and assessed for their ability to enhance metal ion release from silicone elastomers containing copper nanoparticles (CN), zinc nanoparticles (ZN), copper sulfate pentahydrate (CSP) and zinc acetate dihydrate (ZAD). Gelatin, PVP, HPMC, and sucrose have previously been incorporated into experimental silicone elastomer devices (Acartürk and Altug 2001; Morrow et al. 2011; McKay et al. 2012; Pattani et al. 2012; Zhang et al. 2013; Du et al. 2018; McBride, Boyd, et al. 2019; McBride, Malcolm, et al. 2019; Shen et al. 2025). Here, we opted to use smaller silicone elastomer rods (40 mm long × 4 mm cross-sectional diameter) rather than full-size vaginal rings. The impact of the four hydrophilic excipients on the swelling, release rates, and mechanical properties of rods were assessed.

**Table 2.** Molecular weight, source, chemical structural formula, and major applications in pharmaceutics of gelatin, PVP, HPMC, and sucrose in pharmaceutics.

Excipients	Molecular weight (g/mol)	Source	Chemical structural formula	Pharmaceutical application	References
HPMC	50,000–1,250,000	Semisynthetic	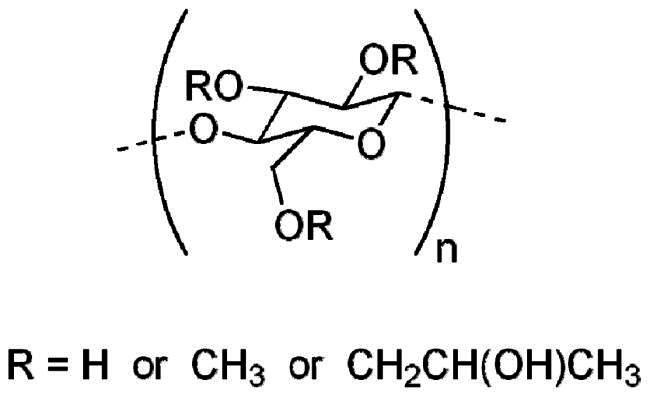	Extended release-matrix former; tablet binder; tablet film coating	Rowe 1977; Chowhan 1980; Shah et al. 1989
PVP	10,000 (average)	Semisynthetic	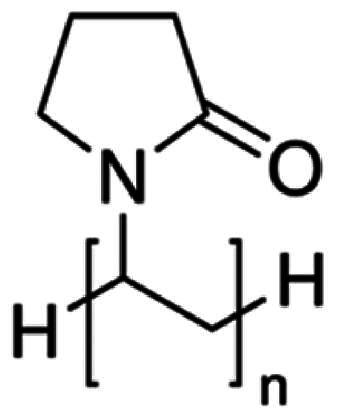	Binder of wet-granulation in tableting; solubilizer in oral and parenteral formulations; coating agents or binders of sugar beads	Iwata and Ueda 1996; Stubberud et al. 1996; Becker et al. 1997
Gelatin	>15,000	Porcine skin	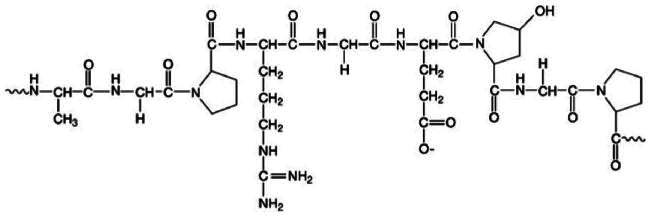	Biodegradable matrix material in an implantable delivery system and capsules	Kommareddy et al. 2003; Mikhailov 2023
Sucrose	342	Plants	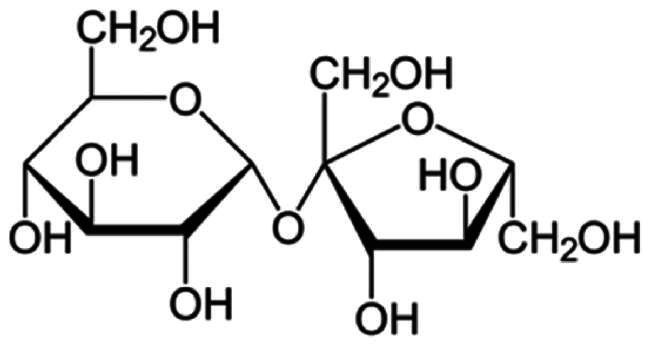	Used as binder in wet-granulation; bulking agent; sweetener in chewable tablets and lozenges; tablet-coating agent	Mullarney et al. 2003; Sugimoto et al. 2006

## 2. Materials and methods

### 2.1. Materials

An unrestricted drug delivery grade addition-cure silicone elastomer (DDU-4320, Parts A and B) was supplied by NuSil Silicone Technology Inc. (Carpinteria, CA, USA). Copper nanopowder (65 nm mean particle size; 99.95%; CN) (Nanografi, Ankara, Turkey) and copper sulfate pentahydrate (≥98.0%; CSP) (Sigma-Aldrich, St. Louis, MO, USA) were selected as sources of Cu^2+^ ions; oxidation/corrosion of copper nanopowder produces cupric ions. Zinc nanopowder (790 nm, 99.95%; Nanografi; 790ZN) and zinc acetate dihydrate powder (Merck KGaA, Darmstadt, Germany; ZAD) were selected as a source of Zn^2+^ ions. Gelatin (from porcine skin), sucrose (>99.5%), and PVP (MW 10,000 g/mol) were purchased from Sigma-Aldrich. HPMC (MW 1261.45 g/mol) was purchased from Alfa Aesar (MA, USA). HPMC and PVP were supplied in micronized form. CSP and ZAD powders were manually milled using a pestle and mortar and sieved using a 100 μm sieve. Polyvinylchloride (PVC) tubing having 4 mm inner CSD was purchased from RS Components Ltd. (Northants, UK). Deionized water was obtained using a Millipore Direct-Q 3 UV Ultrapure Water System (Watford, UK).

### 2.2. Manufacture of silicone elastomer rods

Thirty-six different DDU-4320 matrix-type rod formulations—containing 0 or 5% w/w CN, ZN, CSP, or ZAD combined with 0, 15, or 30% w/w gelatin, PVP, HPMC, and sucrose (Table S1)—were manufactured using a laboratory-scale injection molding machine. Briefly, Part A premixes (10 g) were prepared by accurately weighing the required quantities of actives, excipients, and silicone into screw-cap polypropylene containers and mixing at 3000 rpm for 30 s (DAC-150 FVZ-K SpeedMixer™, Hauschild, Germany) followed by manual mixing for 1 min. Part B premixes (10 g) were similarly prepared. Part A and Part B premixes were combined in a 1:1 ratio, manually mixed for 1 min, and speedmixed (30 s, 3000 rpm). The mixtures were transferred into a cartridge system, injected into the PVC tubing, and cured at 50 °C for 20 h. The silicone elastomer rods were removed from the PVC tubing and cut into 40 mm lengths. Formulation codes (Table 3) adhere to the following convention: the first part of code denotes the API (fixed at 5% w/w loading); the second part of the code denotes the percentage loading of the excipient (15 or 30%) followed by the first letter of the excipient (G for gelatin; P for PVP; H for HPMC and S for sucrose). For example, CSP-15H rods contain 5% w/w CSP and 15% w/w HPMC. B-B represents a rod containing no drug and no excipient (B = blank), while codes that include an API assignment and followed by a B contain active but no excipient.

**Table 3.** Summary of *in vitro* Cu^2+^ ion and Zn^2+^ ion release data for matrix-type silicone elastomer rods containing metal nanopowders + excipients (mean ± *SD*, *n* = 3).

Rods ID	Initial Cu^2+^/Zn loading (µg)	Day 1 release (µg)	Day 8 release (µg)	Day 11 release (µg)	Release percent over 14 days (%)	Higuchi model	
						Release rate (µg/day^1/2^)	*R* ^2^
CN-B	28,540 ± 40	3.51 ± 1.00	0.33 ± 0.47	0.00 ± 0.00	0.06	4.96	0.97
CN-15G	29,417 ± 612	7.86 ± 2.07	2.19 ± 1.08	0.25 ± 0.19	0.17	14.95	0.96
CN-30G	31,108 ± 129	4.96 ± 1.06	6.19 ± 1.35	1.05 ± 0.55	0.28	30.24	0.99
CN-15P	27,940 ± 126	3.80 ± 0.74	1.12 ± 0.59	0.23 ± 0.17	0.11	9.97	0.99
CN-30P	28,135 ± 412	3.09 ± 0.99	3.87 ± 0.82	0.44 ± 0.07	0.15	14.56	1.00
CN-15H	28,435 ± 44	1.20 ± 0.22	1.98 ± 0.41	0.13 ± 0.18	0.08	7.21	0.99
CN-30H	28,835 ± 620	1.08 ± 0.18	2.51 ± 0.20	0.51 ± 0.36	0.09	8.41	0.92
CN-15S	30,353 ± 355	3.76 ± 0.30	1.15 ± 0.31	0.43 ± 0.12	0.08	7.14	0.98
CN-30S	31,271 ± 455	2.64 ± 0.70	1.70 ± 0.76	0.61 ± 0.08	0.11	10.48	0.98
ZN-B	28,095 ± 31	0.26 ± 0.37	0.00 ± 0.00	0.00 ± 0.00	0.00	0.33	0.79
ZN-15G	28,985 ± 212	1.39 ± 0.36	0.65 ± 0.08	0.22 ± 0.31	0.01	0.94	0.91
ZN-30G	30,472 ± 406	4.36 ± 0.41	1.04 ± 0.53	0.08 ± 0.11	0.03	2.23	0.94
ZN-15P	27,778 ± 327	5.50 ± 1.37	0.19 ± 0.27	0.00 ± 0.00	0.03	0.81	0.95
ZN-30P	57,463 ± 391	10.57 ± 1.05	2.00 ± 0.33	0.70 ± 0.38	0.05	7.05	0.99
ZN-15H	28,355 ± 252	1.29 ± 0.36	0.46 ± 0.33	0.07 ± 0.10	0.01	0.86	0.93
ZN-30H	28,243 ± 141	3.98 ± 0.54	0.91 ± 0.20	1.70 ± 1.22	0.06	4.00	0.77
ZN-15S	29,860 ± 730	1.31 ± 0.72	0.33 ± 0.46	0.10 ± 0.14	0.01	0.72	0.90
ZN-30S	32,200 ± 514	2.74 ± 1.04	1.68 ± 0.39	0.41 ± 0.38	0.05	4.76	0.93

### 2.3. Swelling testing

Swelling of rods due to absorption of release medium was assessed in parallel with *in vitro* release testing. On Days 0, 7, 14, 21, 28, and 30 of the release experiment, rods were removed from the tubes (see Section 2.4), and rod dimensions (vernier calipers) and weights (4 dp balance) were measured. Swelling percentages in terms of weight, length, and cross-sectional diameter (CSD) were calculated according to Equations (1)–(3), respectively. For nanopowder formulations, swelling measurements were continued until Day 14.

(1) $$\text{weight swelling}(\%)=(W_{t}-W_{0})/W_{0}\times 100$$

(2) $$\text{CSD}\;\text{swelling}\left(\%\right)=\left(\text{CS}D_{t}-\text{CS}D_{0}\right)/\text{CS}D_{0}\times 100$$

(3) $$\text{length}\text{ swelling }\left(\text{\%}\right)=\left(L_{t}-L_{0}\right)/L_{0}\times 100$$

where *W_t_* = weight at time *t*, *W*_0_ = initial weight, *CSD_t_* = CSD at time *t*, *CSD*_0_ = initial CSD, *L_t_* = length at time *t*, and *L*_0_ = initial length.

### 2.4. In vitro *release testing*

Three rod samples were randomly selected from each formulation batch for release testing. On day 0, individual rods were placed into 13 mL plastic tubes (Sarstedt, Wexford, Ireland) containing 10 mL deionized water (pH 5.2) and stored in a shaking incubator at 37 °C and 60 rpm. The release medium was sampled each weekday and then completely replaced with fresh 10 mL deionized water. No sampling was performed during weekends. Following completion of the release experiment, the rods were placed in a 40 °C oven for 7 days to dry.

### 2.5. Mechanical testing

#### 2.5.1. Shore M hardness testing

**Figure 1.**
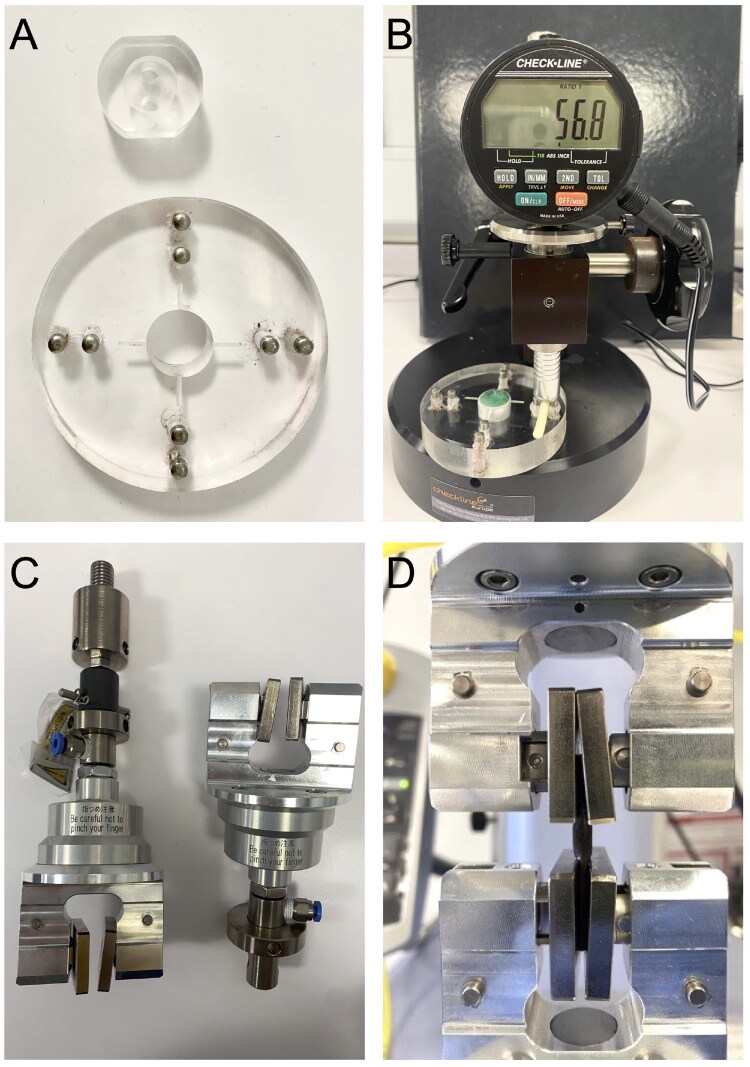
(A) Alignment tools (upper) and 54 × 4 mm holder (lower) for Shore M hardness testing; (B) representative photograph showing a rod placed on the flat for Shore M hardness testing; (C) custom grips for tensile testing of silicone elastomer rods; (D) representative photograph showing a rod placed in the grips for tensile testing.

The Shore M hardness testing of ring formulations was tested according to the material Certificate of Analysis using ASTM D2240 (Type M scale) (‘ASTM D2240-15, Standard Test Method for Rubber Property—Durometer Hardness,’ 2017). For accuracy, the silicone elastomer test materials were required to be flat and of sufficient size (width > 2.5 mm) to ensure edge effects could not influence the measurement. A custom slab mold (dimensions: 54 × 4 mm) was designed and manufactured to enable reproducible sample production (Figure 1(A)). Although rods possess a curved geometry that deviates from ASTM D2240 standard conditions, the Shore M hardness gauge is suitable for such geometries and is frequently employed as an investigational tool in comparative analysis of elastomeric devices (Boyd et al. 2020; Dallal Bashi et al. 2021; Shen et al. 2025).

Rod formulations (after manufacture—D0, post release—D14/D30, and after drying—dried) were tested for Shore hardness using a custom holder (Figure 1(A)) and a Shore M Hardness Tester™ (Checkline RX-DD-M digital durometer Type M) held in an OS-3 stand™ (Checkline Europe, Birmingham, UK; Figure 1(B)) at room temperature (∼20 °C). Before testing, alignment tools (Figure 1(A)) were used to ensure the probe contacted the highest point of the rod sample. Standard test blocks bracketing the hardness range of rings were tested before and after measurement of samples, and the measurements recorded were within the hardness range of the standards, except the hardness value of very few samples dropped <20 after releasing. Four individual measurements were recorded per rod for D0, D14/30, and dried samples. Each hardness value was recorded 5 s after the probe contacted the sample surface.

#### 2.5.2. Tensile testing

Tensile testing was performed on the 36 rod formulations using an Instron Universal Testing System (Instron Model 5564, UK) in tension mode fitted with a 5000 N load cell and Merlin software control. At D0, six rods were randomly selected for each formulation—three were tested immediately, and three were first released into deionized water over 14/30 days and then dried at 40 °C for 7 days before being tested. Rods were placed into the upper and lower grips (Figures 1(C,D)). The initial gauge length was set to 10 mm and then automatically adjusted for each rod to ensure sufficient initial tension. Each rod was stretched using a grip separation speed of 1 N/s until reaching a force of 4 N, and then the speed was changed to 200 mm/min, and the rod stretched until fracture. Elongation (mm) to 4 N (E4N), elongation to break (ELB), and the maximum load (N) at maximum tensile extension were recorded for all rods at D0. The E4N was recorded for all formulations both at D0 and after release/drying. E4N and ELB percentage for individual rod were determined using Equation (4).

(4) $$\text{Elongation }\left(\text{\%}\right)=\frac{\text{max}.\text{ extension at }4N/\text{max}.\text{ load }\left(\text{mm}\right)}{\text{ture gauge length }\left(\text{mm}\right)}\times 100$$

### 2.6. Quantification of copper/zinc ion release

Release of copper and zinc ions was measured using a Shimadzu AA-6300 flame atomic absorption spectrophotometer (Shimadzu, Tokyo, Japan) with a graphite furnace atomizer and an autosampler (ASC-6100, Shimadzu, Tokyo, Japan). Stock solutions of Cu^2+^ or Zn^2+^ ions (500 μg/mL) were prepared by dissolving CSP or ZAD powders in deionized water. Working standard solutions were prepared by dilution of the stock solution with deionized water. Standard and sample solutions were aspirated and absorbance values recorded at 324.8 and 213.9 nm for Cu and Zn, respectively. A copper/zinc hollow-cathode lamp (Mason Technology, Dublin, Ireland) was used as the radiation source and air-acetylene was the carrier gas.

### 2.7. Statistical analysis

Weights and mechanical properties post-manufacture were compared: B-B *versus* each API-B rod formulation; CN-B *versus* each CN rod containing excipient; ZN-B *versus* each ZN rod containing excipient; CSP-B *versus* each CSP rod containing excipient; and ZAD-B *versus* each ZAD rod containing excipient. Weight, CSD, OD, and Shore M hardness values of each rod formulation before IVRT were compared with post IVRT. Additionally, the Shore M and E4N values of the dried formulations were compared with those from the initial manufacturing process. Statistical comparisons were performed using one-way ANOVA analysis by GraphPad Prism software. Significant differences are represented in the figures: * = significant (0.01 < *p* < 0.05), ** = very significant (0.001 < *p* < 0.01), *** = extremely significant (0.0001 < *p* < 0.001), **** = extremely significant (*p* < 0.0001).

## 3. Results and discussion

### 3.1. Visual and physical properties of rods

**Figure 2.**
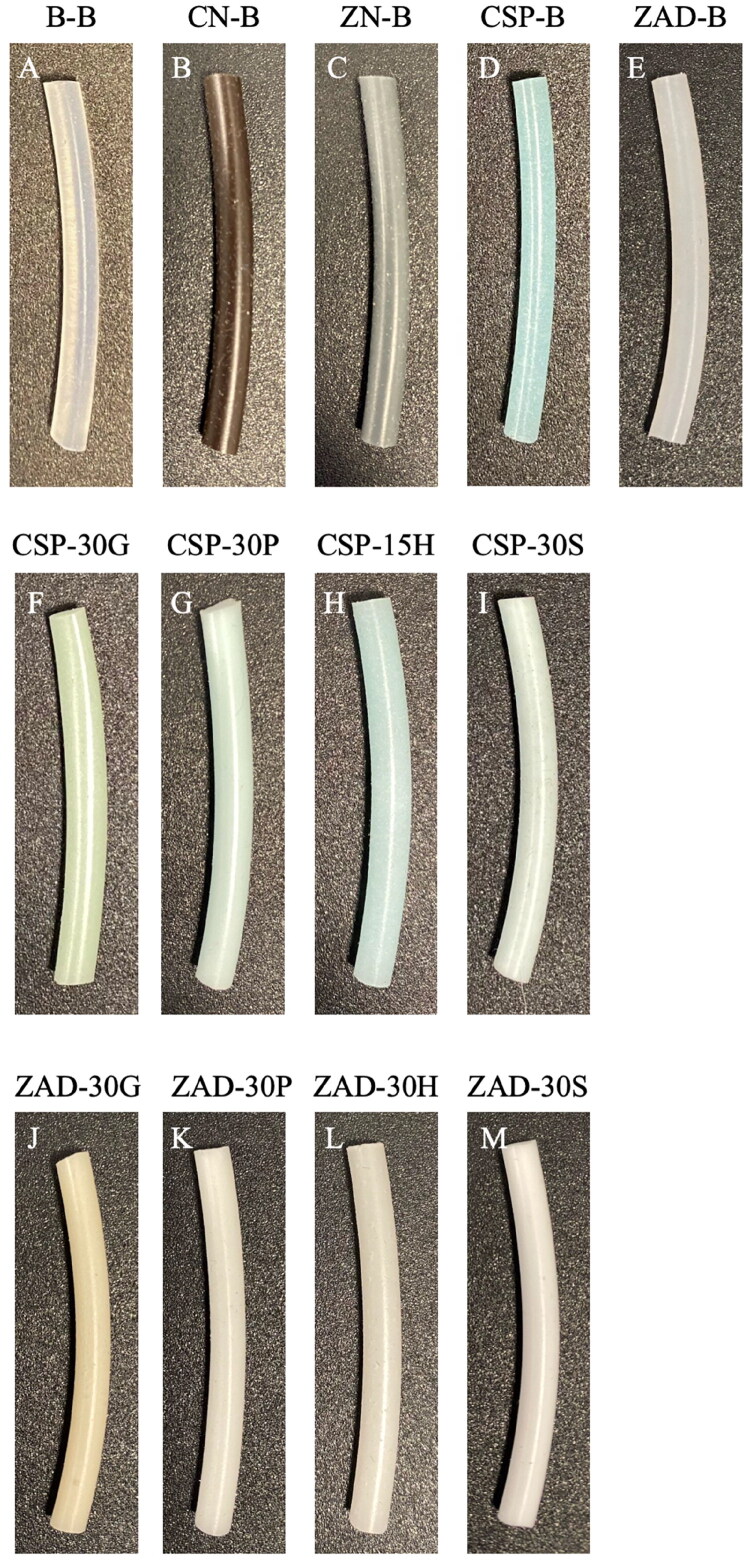
Representative photographs of (A–E) excipient-free, (F–I) CSP with 30% w/w excipients (CSP-30H failed to manufacture, here replaced with CSP-15H), and (J–M) ZAD with 30% w/w excipients rods initially after manufacture.

Representative photographs of rod are shown in Figure 2. Figures S1–S4 provide a more extensive gallery of photographs showing all rods after manufacture, after release, and after drying. All rods were uniform in color, smooth, and free of air bubbles/pockets. The color of the rods reflected the color of the active substances, with some variation in color due to active release and absorption/removal of water.

**Figure 3.**
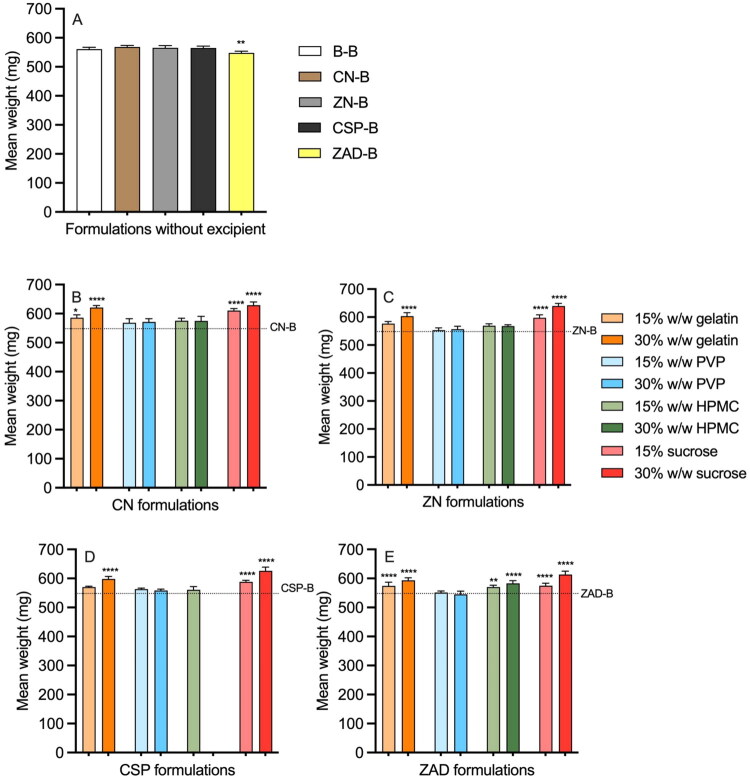
Mean weight for matrix-type (A) excipient-free; (B) CN-excipient; (C) ZN-excipient; (D) CSP-excipient; and (E) ZAD-excipient matrix-type rods post-manufacture. Error bars present the standard deviation of six replicates. (B–E) share the same legend as (C). Dash lines in (B–E) represent the mean weight of rods containing only API. Asterisks (*) above the bars in profile (A) represent the significant difference levels in weight compared with the B-B rod; asterisks (*) above the bars in (B–E) represent the significant difference levels in weight compared with the corresponding excipient-free rods. The absence of an asterisk indicates no significant difference.

Rod weights ranged from 545 to 640 mg due to differences in the types and loadings of both actives and excipients (Figure 3, Table S1). Initial rod dimensions were ∼40 × 4 mm (Table S1). Mean weight of the control B-B rod was 561 mg. The incorporation of 5% w/w ZAD significantly decreased the rod weight, while incorporation of 5% w/w CN, ZN, and CSP showed no difference (Figure 3(A)). The incorporation of 15% w/w or 30% w/w PVP had no significant influence on the weight of any type of rods (Figures 3(B–E)), while inclusion of 15 or 30% w/w sucrose or 30% gelatin significantly increased the rod weights. CSP-30H could not be manufactured successfully, likely due to the high loading of HPMC inhibiting the silicone curing reaction (Malcolm et al. 2016).

### 3.2. Swelling test

**Figure 4.**
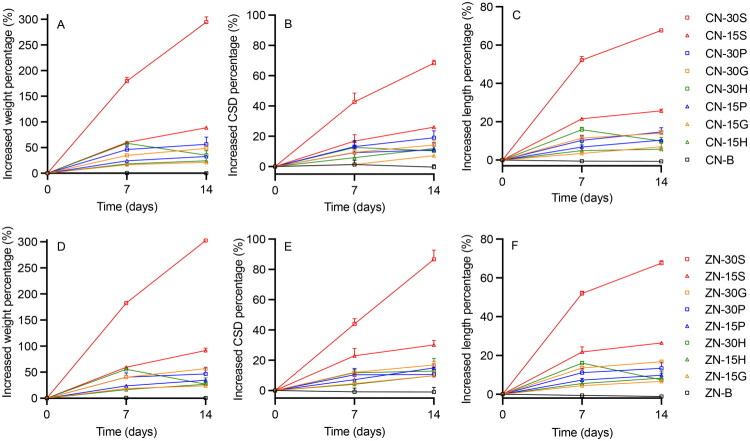
Percentage increases in weight (left), CSD (middle), and length (right) for matrix-type DDU-4320 (A–C) CN-loaded and (D–F) ZN-loaded rods over 14 days. For ease of reading, only upper error bars representing standard deviations of three replicates are shown.

**Figure 5.**
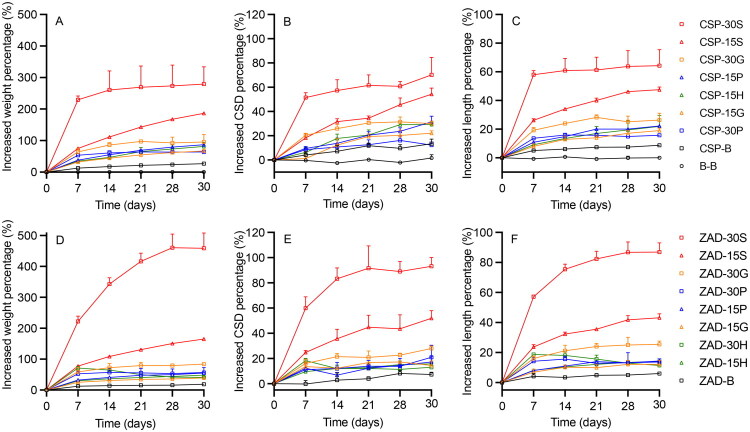
Percentage increases in weight (left), CSD (middle), and length (right) for matrix-type DDU-4320 (A–C) blank and CSP-loaded, and (D–F) ZAD-loaded rods over 30 days. For ease of reading, only upper error bars representing standard deviations of three replicates are shown.

The incorporation of high concentrations of hydrophilic actives and excipients has the potential to cause swelling of silicone elastomer devices due to water/fluid absorption, which is not ideal. For example, swelling of a vaginal ring device could result in involuntary expulsion and/or user discomfort (Efentakis et al. 1997; Johnson et al. 2010; Malcolm et al. 2016; Undie et al. 2020; McLellan-Lemal et al. 2022). The weights, CSDs, and lengths of B-B, CN-B, and ZN-B formulations were largely similar (percentage increases < 1%) before and after *in vitro* release testing, since the silicone elastomer and copper/zinc nanopowders do not absorb water (Figures 4 and 5(A–C)). However, copper/zinc nanopowders are highly reactive and prone to surface oxidation when exposed to moisture (Delalu et al. 2000; Amelkovich et al. 2015). All other formulations—containing various loadings of copper sulfate, zinc acetate, and hydrophilic excipients—showed swelling, and especially with incorporation of 15 or 30% w/w sucrose (Figures 4 and 5). The mean weight, cross-sectional diameter (CSD), and length of CN-30S rods increased 294, 68, and 68%, respectively, and CN-15S rods 89, 26, and 26%, respectively (Figures 4(A–C)). For the other CN + excipient formulations, percentage weight increases were in the range 21–57%, CSD between 7–19%, and length between 6–15%. Similar values and trends were noted for ZN formulations (Figures 4(D–F)). Among the four excipients, nanopowder formulations with 15% gelatin or 15% HPMC showed least swelling, while sucrose formulations showed greatest swelling. With the exception of HPMC, swelling generally increased with higher excipient loadings and longer immersion times. However, for the CN-30H formulation, the rod matrix reached its maximum extent of swelling by Day 7, but then subsequently shrank. This was also observed for ZAD-30H.

The swelling behavior of CSP rod formulations is summarized in Figures 5(A–C). After 30 days, CSP-30S increased significantly in weight, CSD, and length (280, 70, and 193%, respectively), and CSP-15S 186, 54, and 48%, respectively. CSP-B increased ∼27% in weight over 30 days, with minimal changes in dimensions. For all other formulations, weight increased 66–95%, CSD 22–31% and length 16–26%. Similar trends were observed for ZAD formulations (Figures 5(D–F)). Overall, for CSP rods with 15% excipient loadings, rank order of swelling was sucrose > PVP > HPMC > gelatin. For ZAD rods with 30% excipients, swelling rank order was sucrose > gelatin > PVP > HPMC. For CSP and ZAD rods loaded with gelatin and sucrose, the extent of swelling increased with loading. For CSP/ZAD combined with PVP or HPMC, swelling values were similar for both 15 and 30% loadings. In conclusion, among the four excipients, 15% w/w gelatin and 30% w/w PVP showed the least extent of swelling for CSP formulations, while 15% w/w gelatin and 30% w/w HPMC showed the least extent of swelling for ZAD formulations. In the context of silicone elastomer vaginal rings (Boyd et al. 2019), the 10 mL release medium used here was significantly larger than typical vaginal fluid volumes in healthy women (0.5–2 mL) (Owen and Katz 1999), and the rod dimensions were also significantly smaller than normal ring sizes. Without *in vivo* testing, it is difficult to extrapolate these data to predict the extent of swelling of similarly formulated vaginal ring devices.

### 3.3. In vitro *release of copper and zinc ions*

**Figure 6.**
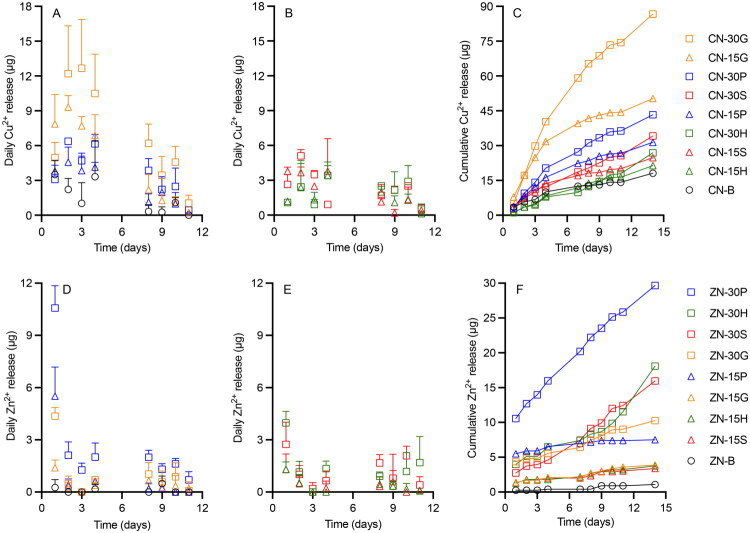
Daily and cumulative release (µg) *versus* time profiles for release of Cu^2+^ (above) and Zn^2+^ (below) ions from matrix type of DDU-4320 (A–C) CN-loaded and (D–F) ZN-loaded rods into deionized water over 14 days, respectively. Upper error bars represent standard deviation of three replicates. (A,B) share the same legend as (C); while (D,E) share the same legend as (F).

Conventional organic small molecule drug substances—which are usually crystalline and micronized—are dispersed uniformly throughout silicone matrix-type drug delivery devices. Upon placement of the device into the release medium, drug on the device surface dissolves in the release medium causing an initial burst release (Malcolm et al. 2003). However, in this study, results from *in vitro* release testing of CN and ZN formulations showed no burst release; release was very low (Figure 6 and Table 3) and often below the LOQ. Among CN formulations (Figures 6(A–C)), CN-30G released the highest daily quantity of Cu^2+^ ions (∼13 µg) on Day 3 and was the only CN formulation able to continually release ions over the two-week test period. For the CN-B formulation, daily Cu^2+^ release was highest (∼3.9 µg) on Day 14 (over weekend), and release was only quantifiable for seven days during the test. However, coformulation of CN with all four excipients significantly increased the quantity and extended the duration of Cu^2+^ release. For ZN-loaded rods, ZN-30P showed the highest daily Zn^2+^ release (∼11 µg) on Day 1 (Figures 6(D–F)); by comparison, the ZN-B formulation maximally released 0.5 µg Zn ions release on Day 9, with no release measured on other days. ZN-30P sustained release of Zn^2+^ over two weeks, while ZN-15G, ZN-15P, and ZN-15S released Zn ions <5 days even with incorporation of excipients.

**Figure 7.**
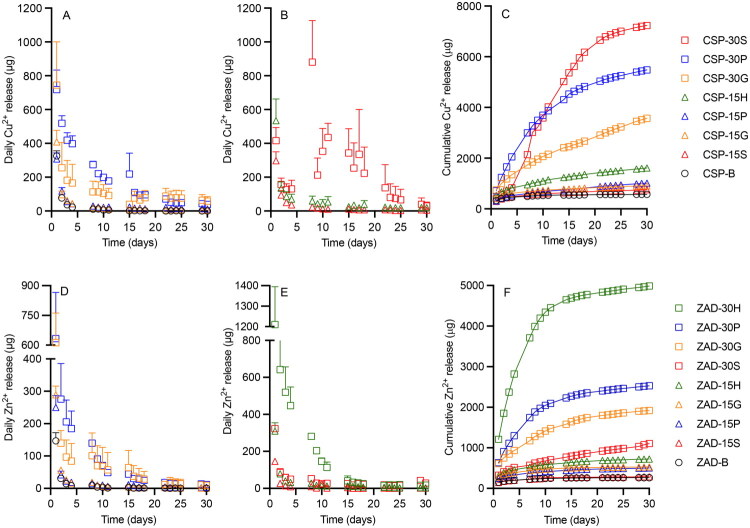
Daily and cumulative release (µg) *versus* time profiles for release of Cu^2+^ (above) and Zn^2+^ (below) ions from matrix type of DDU-4320 (A–C) CSP-loaded and (D–F) ZAD-loaded rods into deionized water over 30 days, respectively. Upper error bars represent standard deviation of three replicates. (A,B) share the same legend as (C); (D,E) share the same legend as (F).

Compared with CN/ZN formulations, release of Cu^2+^ and Zn^2+^ was significantly increased for rods containing the metal salts CSP and ZAD (Figure 7 and Table 4). Except for CSP-30S, burst release was observed for all formulations. On Day 1, Cu^2+^ and Zn^2+^ release ranged from 297–744 µg and 146–1210 µg, respectively, followed by declining release on subsequent days. CSP-30S showed an irregular daily release profile, likely due to swelling of the device, with maximum Cu^2+^ release on Day 8 (880 µg). CSP-30G and ZAD-30H formulations showed the highest Day 1 release—744 µg Cu^2+^ and 1210 µg Zn^2+^ ions, respectively. There were three days when CSP-B released Cu ions quantities less than the LOQ, while CSP-30G released 58 µg Cu^2+^ on Day 30. ZAD-30H released 17 µg Zn^2+^ on Day 30, compared to 0.5 µg Zn^2+^ with ZAD-B. For CSP formulations having 15% w/w excipient loadings, CSP-15H provided the highest Cu^2+^ release (534 µg Day 1, 20 µg Day 30).

**Table 4.** Summary of *in vitro* Cu^2+^ and Zn^2+^ ion release data for matrix-type silicone elastomer rods containing metal salts + excipients (mean ± *SD*, *n* = 3).

Rods ID	Initial Cu^2+^/Zn loading (µg)	Day 1 release (µg)	Day 15 release (µg)	Day 30 release (µg)	Release percent over 30 days (%)	Higuchi model	
						Release rate (µg/day^1/2^)	*R* ^2^
CSP-B	7249 ± 28	326 ± 25	1.7 ± 0.6	0.6 ± 0.9	7.9	43.16	0.83
CSP-15G	7226 ± 18	409 ± 56	6.4 ± 0.6	5.5 ± 0.7	12.6	99.38	0.96
CSP-30G	7564 ± 59	744 ± 209	39.6 ± 11.5	58.1 ± 22.7	47.3	611.2	1.00
CSP-15P	7187 ± 46	308 ± 29	23.5 ± 8.4	11.8 ± 3.8	14.1	148.0	1.00
CSP-30P	7102 ± 73	718 ± 94	218.7 ± 100.5	38.4 ± 4.3	77.2	1397	1.00
CSP-15H	7194 ± 116	534 ± 105	25.2 ± 7.7	20.3 ± 8.0	22.4	236.1	1.00
CSP-15S	7429 ± 34	297 ± 43	11.9 ± 3.0	4.8 ± 2.2	10.1	90.17	0.96
CSP-30S	8023 ± 145	416 ± 63	432.8 ± 117.6	29.4 ± 19.4	90.1	1590	0.91
ZAD-B	8207 ± 88	147 ± 21	3.4 ± 0.2	0.5 ± 0.6	3.2	22.82	0.86
ZAD-15G	8727 ± 41	289 ± 22	4.9 ± 0.9	0.8 ± 0.3	6.1	47.80	0.88
ZAD-30G	8800 ± 107	612 ± 122	64.2 ± 43.0	9.6 ± 5.5	21.8	302.0	0.97
ZAD-15P	8166 ± 69	250 ± 30	11.4 ± 1.5	3.3 ± 1.9	6.2	54.19	0.96
ZAD-30P	7966 ± 36	633 ± 190	44.2 ± 4.6	12.2 ± 0.5	31.8	400.9	0.91
ZAD-15H	8528 ± 110	310 ± 36	12.9 ± 4.1	3.2 ± 1.1	8.5	86.66	0.96
ZAD-30H	8579 ± 122	1210 ± 151	40.8 ± 21.9	17.2 ± 0.8	58.1	1102	0.94
ZAD-15S	8553 ± 74	± 11	3.6 ± 0.8	1.4 ± 0.6	3.4	29.42	0.89
ZAD-30S	9085 ± 237	323 ± 4	25.9 ± 1.7	29.0 ± 1.1	12.2	166.3	1.00

Historically, drug release from simple silicone elastomer matrix-type devices containing hydrophobic small-molecule drugs has been modeled using the Higuchi equation (Equation 5), with certain important assumptions and criteria—(i) drug is uniformly dispersed throughout the matrix, (ii) initial drug concentration in the device is much higher than drug solubility in the polymer matrix (*A* ≫ *C_SIL_*), (iii) drug diffusion occurs in one dimension, (iv) drug diffusion is the rate-limiting step, (v) the drug diffusion coefficient is constant, and (vi) sink conditions are maintained (Higuchi 1961). For most rod formulations tested in this study, criteria (iv) and (v) do not hold since the devices swell due to water uptake during *in vitro* release testing. This is confirmed by coefficient of determination (*R*^2^) values for linear regression analysis of cumulative release *vs* root time (*t^½^*) plots for up to 60% of drug release (Figure S5) that often significantly deviate from 1.0 (Tables 3 and 4). For the purposes of this study, we designate *R*^2^ ≥ 0.99 as good fit, 0.99 > *R*^2^ ≥ 0.97 as moderate fit, and *R*^2^ < 0.97 as poor fit. We understood that high *R*^2^ values might be achieved while still violating Higuchi model assumptions. Nonetheless, the release rates (µg/day^½^) derived from application of the Higuchi model are useful for comparing formulations (Tables 3 and 4).

(5) $$Q=\sqrt{D_{\text{SIL}}(2A-C_{\text{SIL}})C_{\text{SIL}}t}$$

where *Q* is the cumulative amount of drug released per unit area of device (mg/cm^2^), *A* is the drug loading (mg/cm^3^), *C_SIL_* is the solubility of the drug in silicone elastomer (mg/cm^3^), *D_SIL_* is the diffusion coefficient of the drug in the elastomer (cm^2^/day), and *t* is time (days).

Cumulative release of Cu^2+^ from CN formulations ranged from 18 to 87 µg and followed the rank order CN-30G > CN-15G > CN-30P > CN-30S > CN-15P > CN-30H > CN-15S > CN-15H > CN-B (Figure 6(C)). Cumulative Cu^2+^ release and the release rate for CN-30G (87 µg, 30.24 µg/day^½^, respectively) were five times greater than CN-B (18 µg, 4.96 µg/day^½^, respectively). For the Zn nanopowder formulations, rank order of cumulative Zn^2+^ release was ZN-30P (∼30 µg) > ZN-30H > ZN-30S > ZN-30G > ZN-15P > ZN-15G/ZN-15H/ZN-15S > ZN-B (Figure 6(F)). Zn^2+^ cumulative release for ZN-30P was 30 times that of ZN-B (∼1 µg). As for Cu^2+^, Zn^2+^ release rates were significantly enhanced by incorporating 15% or 30% gelatin, PVP, HPMC, and sucrose. 30% w/w PVP provided the largest enhancement (20-fold).

Over the 30-day period, cumulative release of Cu^2+^ ions from CSP formulations ranged from 570 to 7230 µg (Figure 7(C)); rank order was CSP-30S > CSP-30P > CSP-30G > CSP-15H > CSP-15P > CSP-15G > CSP-15S > CSP-B. Cumulative Cu^2+^ release from CSP-30S was more than 12-fold greater than the control CSP-B; further, ∼90% of the original Cu^2+^ loading was released. Release rate of CSP-30S (1590 µg/day^1/2^) was more than 36 times that for CSP-B (43 µg/day^½^) (Table 4). For formulations containing 15% w/w excipient loading, HPMC showed the greatest enhancement of Cu^2+^ release (1614 µg 30 days; 22% w/w released; release rate 236 µg/day^½^). The cumulative release of Zn^2+^ from ZAD formulations ranged from 264–4987 µg, with rank order ZAD-30H > ZAD-30P > ZAD-30G > ZAD-30S > ZAD-15H > ZAD-15G > ZAD-15P > ZAD-15S > ZAD-B (Figure 7(F)). Cumulative Zn^2+^ release from ZAD-30H (58% Zn^2+^ released, 1102 µg/day^½^) was significantly greater than for ZAD-B (3% Zn^2+^ released, 27 µg/day^1/2^). With the inclusion of excipients, all metal salts formulations were able to maintain the release of Cu/Zn ions over one month.

Although deionized water was used as the release medium in this study, we acknowledge that it does not fully mimic the physiological environment of the vagina, which typically contains 0.5–2 mL of acidic fluid with pH ∼4.2 and moderate ionic strength (Owen and Katz 1999). The use of 10 mL deionized water was a deliberate simplification, selected to maintain sink. This approach is commonly adopted in early-stage screening studies to facilitate comparative evaluation of excipient performance (Boyd et al. 2019). We anticipate that under physiological conditions, the release of metal ions is expected to be slower than observed *in vitro*. This is primarily due to the limited fluid volume in the vaginal environment, which reduces water uptake into the matrix and slows the dissolution of metal salts. In addition, the presence of biological components (such as ions and proteins) may lead to complexation with Cu^2+^ and Zn^2+^, which can further delay the diffusion of free metal ions. Together, these factors suggest that *in vivo*, the formulations may exhibit more gradual and sustained ion release, which could be beneficial for prolonged local spermicidal activity while minimizing systemic exposure. The formulation screening conducted here provides an important foundation for subsequent *in vitro* or *in vivo* studies under more physiologically relevant conditions, such as simulated vaginal fluid or human cervicovaginal secretions.

#### 3.3.1. *Mathematical modeling of* in vitro *release data*

To better accommodate device swelling, *in vitro* release data was also modeled using the Korsmeyer-Peppas equation (Equation 6) (Peppas 1985). For cylindrical devices, a release exponent value (*n*) of 0.45 is indicative of drug release that obeys Fickian diffusion, for which the rate of drug diffusion is much smaller than the relaxation time of the polymer chains (Crank 1975; Fu and Kao 2010; Talevi and Ruiz 2021). When using the equation to analyze drug release from porous systems, the value of *n* may be <0.45. This is referred to as quasi-Fickian diffusion and occurs when additional mechanisms influence the release of actives, such as diffusion through a swollen matrix comprising water-filled pores (as is likely occurring here). *n* values between 0.45 and 0.89 indicate deviation from Fickian diffusion, are classified as anomalous (non-Fickian), and are common when both swelling and diffusional process influence drug release. According to this model, *n* values equal to 0.89 are classified as case-II transport (zero-order release), for which the diffusional processes are much faster than the polymer relaxation process, and drug release is mainly controlled by swelling. Super case II transport—where *n* > 0.89—is rarely observed; it is indicative of accelerated release of the dissolved drug from the matrix (Bruschi 2015).

(6) $$M_{t}/M_{\infty }=K\;t^{n}$$

where *M_t_/M_∞_* is a fraction of drug released at time *t*, *K* is the release rate constant, and *n* is the release exponent.

**Figure 8.**
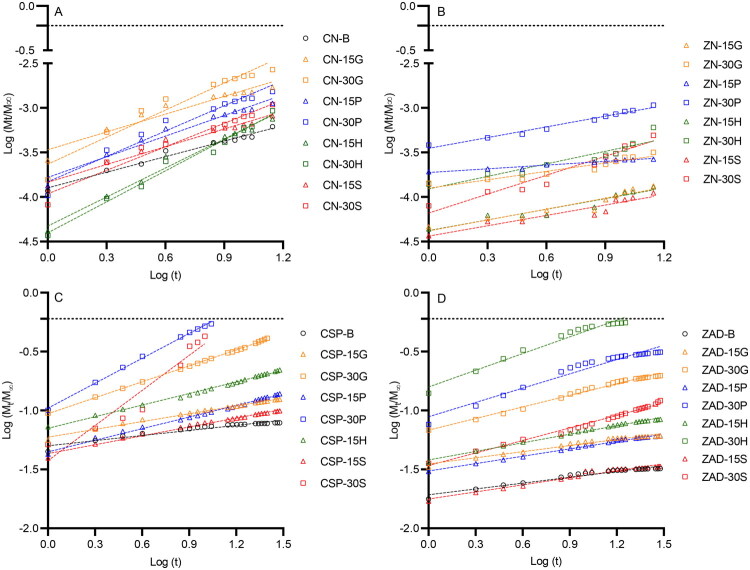
Graphs showing log fraction Cu^2+^/Zn^2+^ release *versus* log *t* (days) for matrix-type rods containing (A) CN, (B) ZN, (C) CSP, and (D) ZAD.

Graphs of log fraction Cu^2+^/Zn^2+^ release *versus* log time from the various rods are presented in Figure 8. Exponent values for CN formulations (Table 5) were larger than 0.45 (anomalous transport), while most ZN, CSP, and ZAD formulations had *n* < 0.45 (quasi-Fickian diffusion). Release for ZN-B could not be modeled using the Korsmeyer-Peppas equation as cumulative release was too low. For copper sulfate and zinc acetate formulations (Table 5), CSP-30G showed Fickian release (*n* = 0.45, release proportional to *t*^½^). CSP-30P and ZAD-30H showed anomalous (non-Fickian) release with *n* values between 0.45 and 0.89. CSP-30S was the only metal salt formulation that released according to a super-case II transport mechanism. For other metal salts formulations, quasi-Fickian drug release was observed (*n* < 0.45).

**Table 5.** Analysis of Cu^2+^ and Zn^2+^ ion release from matrix-type rods with Korsmeyer-Peppas model (limited to the first 60% of drug release).

Rod ID	*n*	Drug transport mechanism	*R* ^2^	Rod ID	*n*	Drug transport mechanism	*R* ^2^
CN-B	0.583	Anomalous	0.980	CSP-B	0.144	Quasi-Fickian	0.928
CN-15G	0.665	Anomalous	0.934	CSP-15G	0.221	Quasi-Fickian	0.992
CN-30G	1.012	Super case-II	0.942	CSP-30G	0.451	Fickian	0.999
CN-15P	0.772	Anomalous	0.968	CSP-15P	0.328	Quasi-Fickian	0.994
CN-30P	0.948	Super case-II	0.951	CSP-30P	0.705	Anomalous	0.998
CN-15H	1.086	Super case-II	0.983	CSP-15H	0.328	Quasi-Fickian	0.995
CN-30H	1.160	Super case-II	0.986	CSP-15S	0.251	Quasi-Fickian	0.988
CN-15S	0.665	Anomalous	0.970	CSP-30S	0.993	Super case-II	0.923
CN-30S	0.879	Anomalous	0.964	ZAD-B	0.165	Quasi-Fickian	0.953
ZN-B	N/A	N/A	N/A	ZAD-15G	0.173	Quasi-Fickian	0.965
ZN-15G	0.407	Quasi-Fickian	0.895	ZAD-30G	0.328	Quasi-Fickian	0.989
ZN-30G	0.314	Quasi-Fickian	0.898	ZAD-15P	0.209	Quasi-Fickian	0.995
ZN-15P	0.139	Quasi-Fickian	0.961	ZAD-30P	0.404	Quasi-Fickian	0.954
ZN-30P	0.400	Quasi-Fickian	0.978	ZAD-15H	0.243	Quasi-Fickian	0.993
ZN-15H	0.397	Quasi-Fickian	0.923	ZAD-30H	0.473	Anomalous	0.968
ZN-30H	0.469	Anomalous	0.870	ZAD-15S	0.200	Quasi-Fickian	0.962
ZN-15S	0.387	Quasi-Fickian	0.899	ZAD-30S	0.354	Quasi-Fickian	0.990
ZN-30S	0.706	Anomalous	0.948				

*n* and *R*^2^ values are rounded to 3 dp.

**Figure 9.**
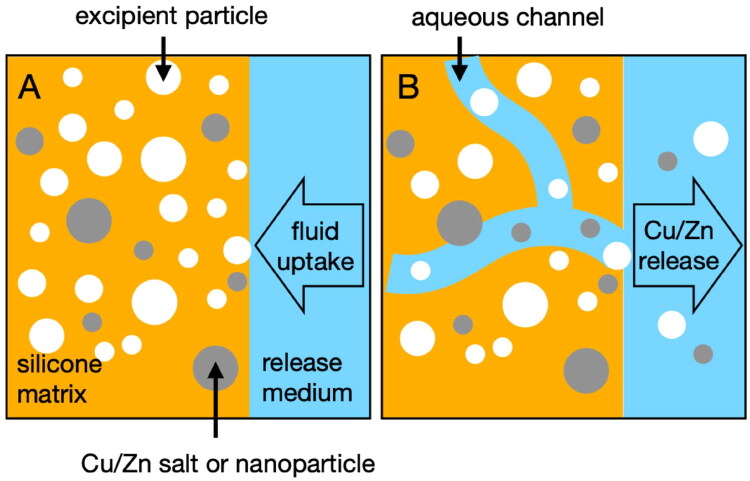
Release of copper/zinc ions (whether incorporated as a salt or a nanoparticle) from a silicone elastomer matrix containing a hydrophilic excipient is facilitated by fluid update into the matrix (A) and the subsequent formation of aqueous channels throughout the matrix. This figure has been adapted from a previously published figure (Di Colo 1992).

The limited release of Cu^2+^/Zn^2+^ from nanopowder formulations is a consequence of their poor solubility and diffusion within the hydrophobic silicone matrix (Malcolm et al. 2003) Incorporating hydrophilic excipients, such as gelatin, PVP, HPMC, and sucrose into the silicone elastomer promotes the formation of aqueous pores, channels, and cracks when immersed in the release medium. As these excipients swell and leach out, they progressively increase fluid penetration and expose the embedded nanopowder and salts, thereby enhancing ion diffusion and release. This observation is consistent with the biphasic release mechanism first described by Colo in 1992 (Figure 9) (Di Colo 1992). According to this model, the first stage involves a diffusion-controlled process of Cu^2+^/Zn^2+^ ions fully surrounded by the pore fluid and then released; while the second phase involves a combined dissolution–diffusion-controlled mechanism of Cu^2+^/Zn^2+^ ions embedded within or partially enclosed by the silicone elastomer. To validate this biphasic behavior, scanning electron microscopy was performed on the CSP-30S formulation, which achieved over 90% release of CSP. Cross-sectional images (Figure S6, Supplementary Material) revealed a porous matrix devoid of residual particles after drying post-release testing, confirming the structural changes in the matrix between before- and post-release.

### 3.4. Mechanical testing

#### 3.4.1. Shore M hardness test

**Figure 10.**
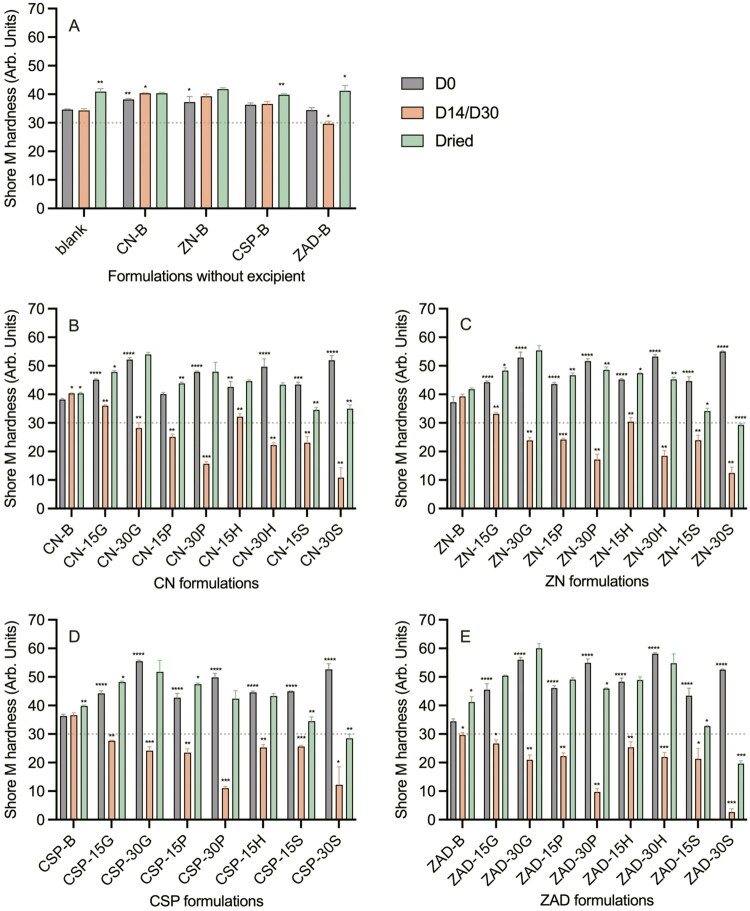
Mean Shore M hardness values for matrix-type rod formulations at D0, D14/D30, and Dried: (A) excipient-free; (B) CN; (C) ZN; (D) CSP; and (E) ZAD. Error bars represent standard deviation of three replicates. The dashed line at 30 represents the Shore M hardness value of the Annovera^®^ vaginal ring. Shore M hardness values of all rod formulations were smaller than that of NuvaRing^®^ (87 units). Thus, Shore M hardness values >30 fall within the range of marketed vaginal rings. The asterisks above purple bars present significant difference levels in Shore M hardness values between B-B rod and API-B rod/between API-B and API-excipient rod at D0; asterisks above orange bars show significant differences for each rod between D0 and D14/30; while asterisks above green bars indicate differences for each rod between D0 and Dried. There.

Since many of the rod formulations swelled when immersed in water/fluid, it was deemed important to assess changes in their hardness. For reference, the Shore M hardness values for four commercial vaginal rings—Annovera^®^, Progering^®^, Estring^®^, and NuvaRing^®^—have previously been reported as 30, 42, 51, and 87, respectively (Shen et al. 2025). The Shore M hardness values of the various rod formulations—measured soon after manufacture (D0), immediately after completion of *in vitro* release testing (D14/D30), and after drying post-release (Dried)—ranged between 2.6 and 60.1 (Figure 10). While hardness values for CN, ZN, CSP, and ZAD formulations containing no excipients fell within a relatively narrow range (29.7–41.7; indicating only minor changes in hardness pre/during/post testing), formulations containing excipients invariably showed a V-shaped trend in hardness, with values relatively high post-manufacture, decreasing significantly following measurement immediately after completion of *in vitro* release testing, and then fully or partially recovering with subsequent oven drying to evaporate the absorbed water (Figures 10(B–E)). Immediately post-release (D14/D30; before drying), only certain CN or ZN formulations—specifically, CN-B, ZN-B, CSP-B, CN-15G, ZN-15G, CN-15H, and ZN-15H—fell within the hardness range of commercial rings; for all other excipient-loaded rod formulations, water uptake and swelling reduced hardness values below 30. Several notable trends were observed—(i) for D0 samples, hardness values increased with increasing excipient loading (0 < 15 < 30%), presumably due to a mechanical filler effect; (ii) for D14/D30 samples (post release testing), hardness values decreased with increasing excipient loading (0 > 15 > 30%); and (iii) for D14/D30 samples, hardness values generally followed the excipient rank order S < P < H/G, correlating with the absolute solubilities and dissolution rates of the excipients.

#### 3.4.2. Elongation testing

**Figure 11.**
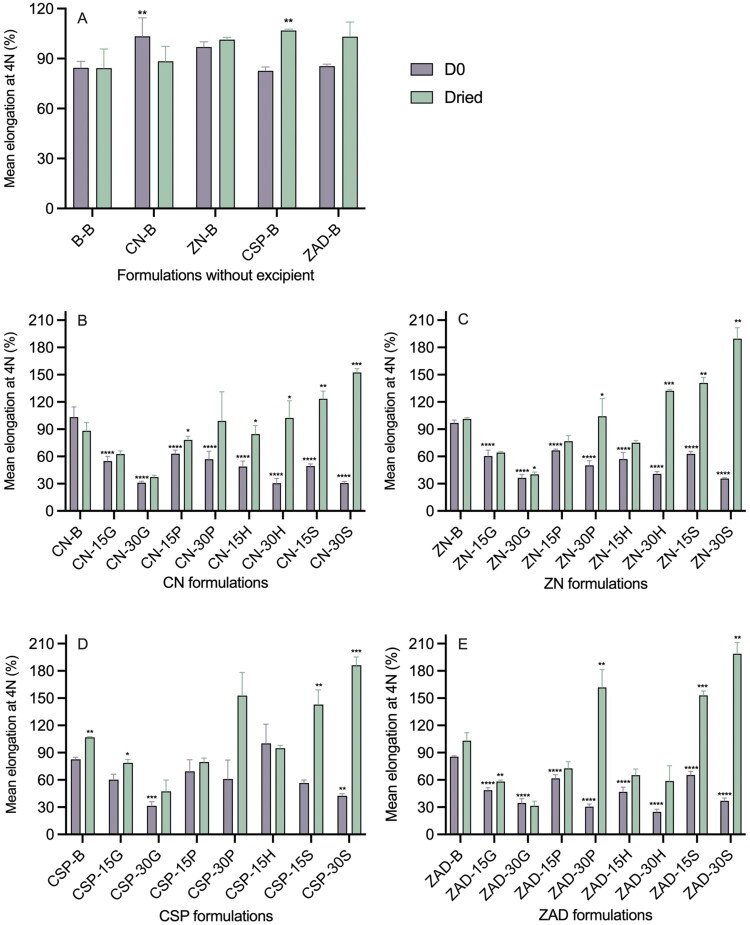
Mean elongation at 4 N for (A) excipient-free; (B) CN; (C) ZN; (D) CSP; and (E) ZAD matrix-type rods at D0 and Dried. Error bars represent the standard deviation of three replicates. The asterisks above purple bars present significant difference levels of elongation of 4 N between B-B rod and API-B rods/between API-B and API-excipient rods at D0; asterisks above green bars present significant difference levels of elongation of 4 N of each rod at D0 *versus* Dried.

Elongation testing at 4 N was performed at two timepoints—D0 and Dried; results are presented in Figure 11. For reference, blank silicone elastomer control rods had D0 and Dried elongation values of ∼84%. For formulations containing 5% w/w of the various actives but no excipients (Figure 11(A)), mean percentage elongations (ranging from 83 to 107%) were mostly not significantly different from the B-B control. However, as with Shore hardness measurements, incorporation of excipients significantly modified the elongation characteristics (Figures 11(B–E)). For D0 samples, elongation values for excipient-loaded formulations were generally lower than for the excipient-free controls. For Dried samples, elongation values were dependent upon the excipient type and loading; notably, gelatin produced the lowest values (31% for ZAD-30G Dried), while sucrose produced values higher than controls (maximum elongations ∼200% measured for 30% sucrose).

**Figure 12.**
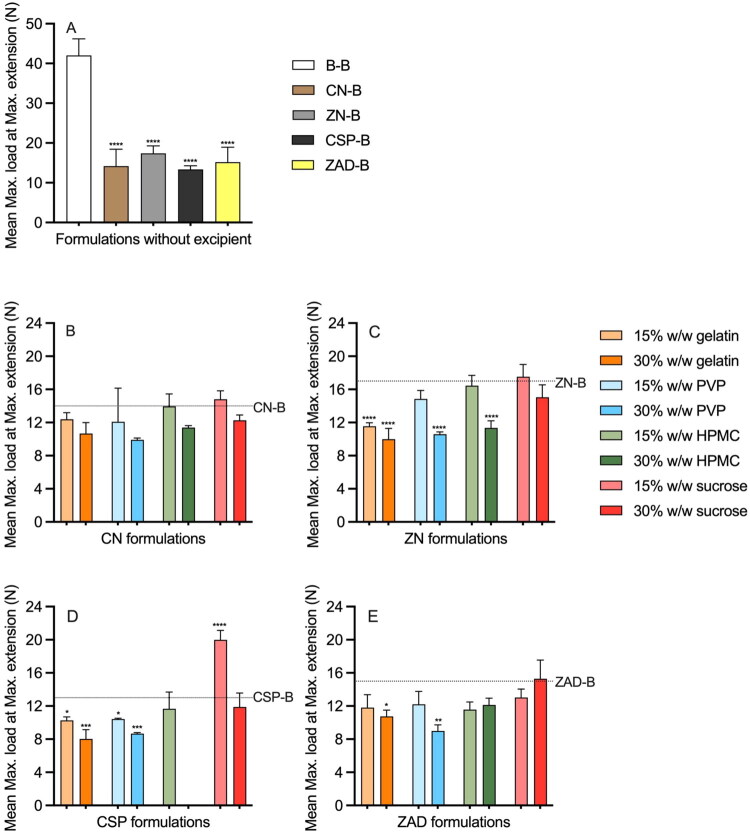
Mean maximum load at maximum extension for (A) excipient-free; (B) CN-excipient; (C) ZN-excipient; (D) CSP-excipient; and (E) ZAD-excipient matrix-type rods post-manufacture. Error bars represent standard deviations of three replicates. (B–E) share the same legend as (C). Dash lines in (B–E) represent the mean maximum load of rods containing only API. Asterisks above the bars in (A) represent the significant difference levels in maximum load compared with the B-B rod; asterisks above the bars in (B–E) represent the significant difference levels in maximum load compared with the corresponding excipient-free rods.

‘Maximum load at maximum extension’—a measure of how much force a material or device can withstand at its most stretched state—is a useful indicator of mechanical durability and robustness. Values for mean maximum load at maximum extension at D0 were very significantly lower for all excipient-free active formulations compared to the 42 N measured for the non-active control (Figure 12(A)). Incorporation of excipients generally caused the maximum load to decrease compared to active-only formulations, with the notable exception of CSP-15S (Figures 12(B–E)).

**Figure 13.**
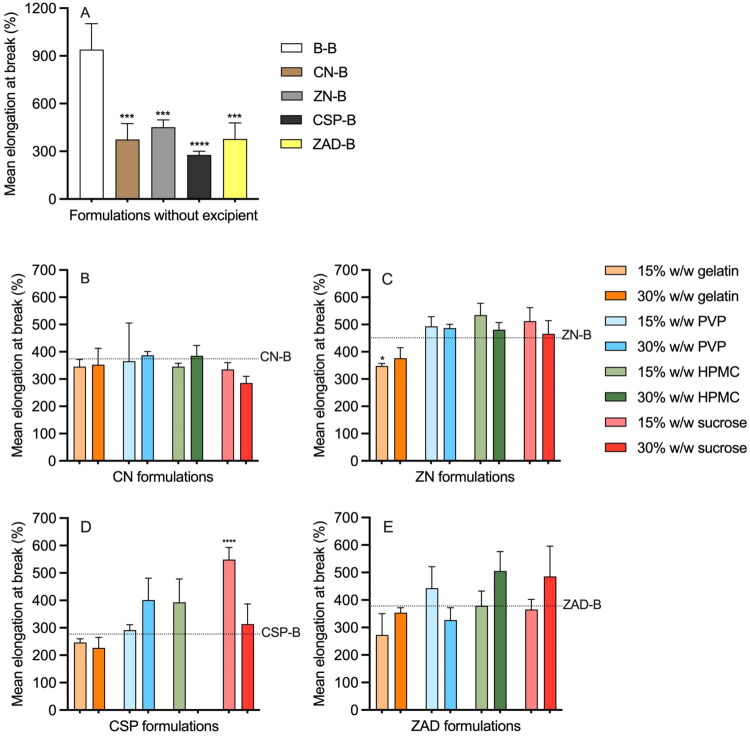
Mean elongation at break (%) for (A) excipient-free; (B) CN-excipient; (C) ZN-excipient; (D) CSP-excipient; and (E) ZAD-excipient matrix-type rods at post-manufacture. Error bars represent the standard deviation of three replicates. (B–E) share the same legend as (C). Dash lines in (B–E) represent the %ELB of rods containing only API. Asterisks (*) above the bars in (A) represent the significant difference levels in %ELB compared with the B-B rod; asterisks above the bars in (B–E) represent the significant difference levels in %ELB compared with the corresponding excipient-free rods.

‘Percentage elongation at break’ (%ELB) is a mechanical/tensile property that describes how much a material can stretch before it breaks. This parameter has been widely reported for vaginal rings (Boyd et al. 2020; Dallal Bashi et al. 2021); for example, ELB% values for Femring^®^, NuvaRing^®^, and Estring^®^ were previously measured as 263, 824, and 1511%, respectively (McCoy et al. 2019), indicating the very different properties of these rings. Mean %ELB values for all rod formulations are presented in Figure 13. Compared to the 939% ELB value for the B-B control rod, values were decreased significantly following incorporation of actives: ZN-B (451%) > ZAD-B (378%) > CN-B (374%) > CSP-B (277%) (Figure 12(A)). %ELB values for CN and ZN formulations containing excipients ranged from 285 to 385 and 348 to 535%, respectively (Figures 12(B,C)). ELB values of 183–548 and 273–485% were measured for CSP and ZAD formulations with excipients, respectively (Figures 13(D,E)). Overall, ELB values for these formulations fell within the range of those measured previously for commercial rings, except for CSP-15G and CSP-30G.

In summary, for D0 rods, mechanical performance is dependent upon the formulation composition. Variations in mechanical properties between D0 and D14/D30 are mostly attributed to water absorption by the rods during *in vitro* release testing, with further contributions due to post-curing effects. Post drying, the compositions of the formulations and the post-curing effect collectively impact the mechanical properties of the dried formulations. For those rods that swelled excessively during *in vitro* release testing, mechanical performance deteriorated significantly.

## 4. Conclusions

The results demonstrate several key findings: (i) copper/zinc nanopowders and salts can be successfully incorporated into addition-cure silicone elastomers; (ii) *in vitro* release of Cu^2+^/Zn^2^ ions from silicone elastomers loaded with divalent salts was ∼100 times greater than when loaded with copper/zinc nanopowders, reflecting the differences in aqueous solubility; (iii) incorporation of four common pharmaceutical excipients—gelatin, polyvinylpyrrolidone, sucrose and hydroxypropyl methylcellulose—at 15 or 30% w/w loadings into silicone elastomer devices containing 5% w/w copper/zinc nanopowders or salts significantly enhanced the release of Cu^2+^/Zn^2^ ions (up to ∼30-fold); (iv) the enhanced release is due to water absorption into the silicone elastomer devices, causing swelling of the devices to an extent proportional to the excipient loading; (v) water absorption—as measured by weight increase—was greatest for devices containing sucrose, the most water-soluble of the four excipients tested; (vi) the extent of increase in *in vitro* release of Cu^2+^/Zn^2+^ was highly dependent on the type of excipient—for each of copper nanopowders, zinc nanopowders, copper sulfate and zinc acetate, the greatest increase was measured for 30% gelatin, 30% polyvinylpyrrolidone, 30% sucrose, and 30% hydroxypropyl methylcellulose, respectively; and (vii) the incorporation of excipients greatly impacted the mechanical characteristics of the silicone elastomer devices.

The results are significant in the context of ongoing efforts to extend the utility of silicone elastomers to the formulation and release of hydrophilic actives. Currently, all marketed silicone elastomer drug delivery products contain relatively hydrophobic and potent drug actives; hydrophilic drugs do not generally have sufficient solubility within the silicone matrix to release in quantities that are clinically effective. This study demonstrates that while inclusion of hydrophilic excipients may be effective in enhancing release of hydrophilic actives [and presumably also hydrophobic actives (Woolfson et al. 2006)], other issues and challenges are encountered, not least swelling of the devices and changes in their mechanical performance. Certain routes of drug delivery and clinical indications may be able to accommodate such changes, within limits. For example, although marketed silicone elastomer vaginal rings are not known to swell during clinical use, a small increase in weight/dimensions might be clinically acceptable—there are significant differences in weights and dimensions of marketed rings (Boyd et al. 2020; Carson et al. 2021) and considerable variations in the baseline dimensions of the undistended human vagina (Barnhart et al. 2006).

Finally, release of metal ions from silicone elastomers has potential in a range of industrial and clinical indications, due to their antimicrobial, catalytic, and therapeutic properties. While the work here is primarily set within the content of developing vaginal devices for non-hormonal contraception, we can envisage applications around antimicrobial medical devices, bone regeneration and orthopedic implants, cancer therapy, water purification, catalytic applications in chemical processes, and silicone coatings for fabrics releasing antimicrobial ions.

## Supplementary Material

Supplymentary material v2.docx

## Data Availability

The data that support the findings of this study are available from the corresponding author, [R.K.M.], upon reasonable request.
