# Supplementary material for: Use of hydrophilic pharmaceutical excipients to modulate release of metal ions from silicone elastomers
Source: Drug Deliv. 2025 Aug 21;32(1):2545515. doi: 10.1080/10717544.2025.2545515 (PMC12372501; doi:10.1080/10717544.2025.2545515)
Supplement: Supplymentary material v2.docx [file IDRD_A_2545515_SM2985.docx]

**Table S1.** API and excipients compositions for 36 types of matrix-type DDU-4320 rods. Weight, CSD and length values for 36 matrix-type DDU-4320 silicone elastomer rods following manufacture are shown as mean values ± SD (n=6). The standard deviations associated with measurement of rod CSD and length are primarily due to variations associated with use of the vernier callipers.

| Rod formulation | Type and percentage loading of API | Type and percentage loading of excipient | Initial weight (mg) | Initial CSD  (mm) | Initial length  (mm) |
| --- | --- | --- | --- | --- | --- |
| B-B | - | - | 560.7 ± 6.5 | 4.01 ± 0.03 | 40.28 ± 0.12 |
| CN-B | 5% w/w CN | - | 568.2 ± 5.3 | 4.01 ± 0.09 | 39.66 ± 0.32 |
| CN-15G | 5% w/w CN | 15% w/w gelatin | 586.0 ± 10.1 | 4.06 ± 0.05 | 40.05 ± 0.51 |
| CN-30G | 5% w/w CN | 30% w/w gelatin | 621.0 ± 6.7 | 4.11 ± 0.05 | 40.42 ± 0.14 |
| CN-15P | 5% w/w CN | 15% w/w PVP | 568.2 ± 14.3 | 4.08 ± 0.11 | 40.35 ± 0.56 |
| CN-30P | 5% w/w CN | 30% w/w PVP | 571.2 ± 11.0 | 4.08 ± 0.05 | 40.38 ± 0.37 |
| CN-15H | 5% w/w CN | 15% w/w HPMC | 575.3 ± 9.1 | 4.00 ± 0.04 | 39.88 ± 0.41 |
| CN-30H | 5% w/w CN | 30% w/w HPMC | 574.8 ± 16.0 | 4.09 ± 0.09 | 39.97 ± 0.92 |
| CN-15S | 5% w/w CN | 15% w/w sucrose | 610.4 ± 6.9 | 4.11 ± 0.03 | 40.36 ± 0.28 |
| CN-30S | 5% w/w CN | 30% w/w sucrose | 629.2 ± 11.1 | 4.03 ± 0.08 | 40.04 ± 0.52 |
| ZN-B | 5% w/w ZN | - | 565.3 ± 7.9 | 3.98 ± 0.09 | 39.97 ± 0.55 |
| ZN-15G | 5% w/w ZN | 15% w/w gelatin | 576.6 ± 7.5 | 3.97 ± 0.06 | 40.25 ± 0.43 |
| ZN-30G | 5% w/w ZN | 30% w/w gelatin | 603.5 ± 12.2 | 4.04 ± 0.05 | 39.82 ± 0.66 |
| ZN-15P | 5% w/w ZN | 15% w/w PVP | 553.2 ± 8.1 | 4.00 ± 0.06 | 39.90 ± 0.40 |
| ZN-30P | 5% w/w ZN | 30% w/w PVP | 556.9 ± 10.3 | 4.01 ± 0.07 | 40.46 ± 0.80 |
| ZN-15H | 5% w/w ZN | 15% w/w HPMC | 569.2 ± 6.7 | 4.09 ± 0.08 | 39.49 ± 0.45 |
| ZN-30H | 5% w/w ZN | 30% w/w HPMC | 567.8 ± 4.9 | 3.98 ± 0.09 | 39.72 ± 0.28 |
| ZN-15S | 5% w/w ZN | 15% w/w sucrose | 597.7 ± 10.6 | 4.00 ± 0.08 | 39.80 ± 0.35 |
| ZN-30S | 5% w/w ZN | 30% w/w sucrose | 639.6 ± 9.4 | 4.00 ± 0.04 | 40.20 ± 0.41 |
| CSP-B | 5% w/w CSP | - | 564.7 ± 6.9 | 4.03 ± 0.05 | 40.36 ± 0.33 |
| CSP-15G | 5% w/w CSP | 15% w/w gelatin | 570.2 ± 2.7 | 4.04 ± 0.05 | 40.24 ± 0.12 |
| CSP-30G | 5% w/w CSP | 30% w/w gelatin | 598.0 ± 9.2 | 4.01 ± 0.07 | 40.43 ± 0.27 |
| CSP-15P | 5% w/w CSP | 15% w/w PVP | 563.4 ± 3.4 | 4.02 ± 0.04 | 40.48 ± 0.25 |
| CSP-30P | 5% w/w CSP | 30% w/w PVP | 558.8 ± 4.3 | 4.04 ± 0.03 | 40.52 ± 0.36 |
| CSP-15H | 5% w/w CSP | 15% w/w HPMC | 560.6 ± 11.3 | 3.99 ± 0.06 | 40.30 ± 0.62 |
| CSP-15S | 5% w/w CSP | 15% w/w sucrose | 588.4 ± 5.4 | 4.08 ± 0.05 | 39.89 ± 0.38 |
| CSP-30S | 5% w/w CSP | 30% w/w sucrose | 626.1 ± 13.0 | 4.02 ± 0.06 | 40.06 ± 0.53 |
| ZAD-B | 5% w/w ZAD | - | 548.0 ± 5.7 | 4.02 ± 0.08 | 40.12 ± 0.24 |
| ZAD-15G | 5% w/w ZAD | 15% w/w gelatin | 574.4 ± 12.5 | 3.98 ± 0.05 | 40.23 ± 0.28 |
| ZAD-30G | 5% w/w ZAD | 30% w/w gelatin | 593.3 ± 8.9 | 4.00 ± 0.05 | 40.12 ± 0.50 |
| ZAD-15P | 5% w/w ZAD | 15% w/w PVP | 551.5 ± 5.2 | 4.04 ± 0.06 | 39.90 ± 0.29 |
| ZAD-30P | 5% w/w ZAD | 30% w/w PVP | 544.9 ± 11.4 | 4.03 ± 0.07 | 40.42 ± 0.48 |
| ZAD-15H | 5% w/w ZAD | 15% w/w HPMC | 570.2 ± 6.6 | 4.04 ± 0.06 | 40.19 ± 0.19 |
| ZAD-30H | 5% w/w ZAD | 30% w/w HPMC | 582.8 ± 9.5 | 4.08 ± 0.06 | 40.35 ± 0.49 |
| ZAD-15S | 5% w/w ZAD | 15% w/w sucrose | 575.0 ± 8.5 | 4.04 ± 0.04 | 40.06 ± 0.36 |
| ZAD-30S | 5% w/w ZAD | 30% w/w sucrose | 613.2 ± 12.1 | 4.06 ± 0.08 | 40.03 ± 0.32 |


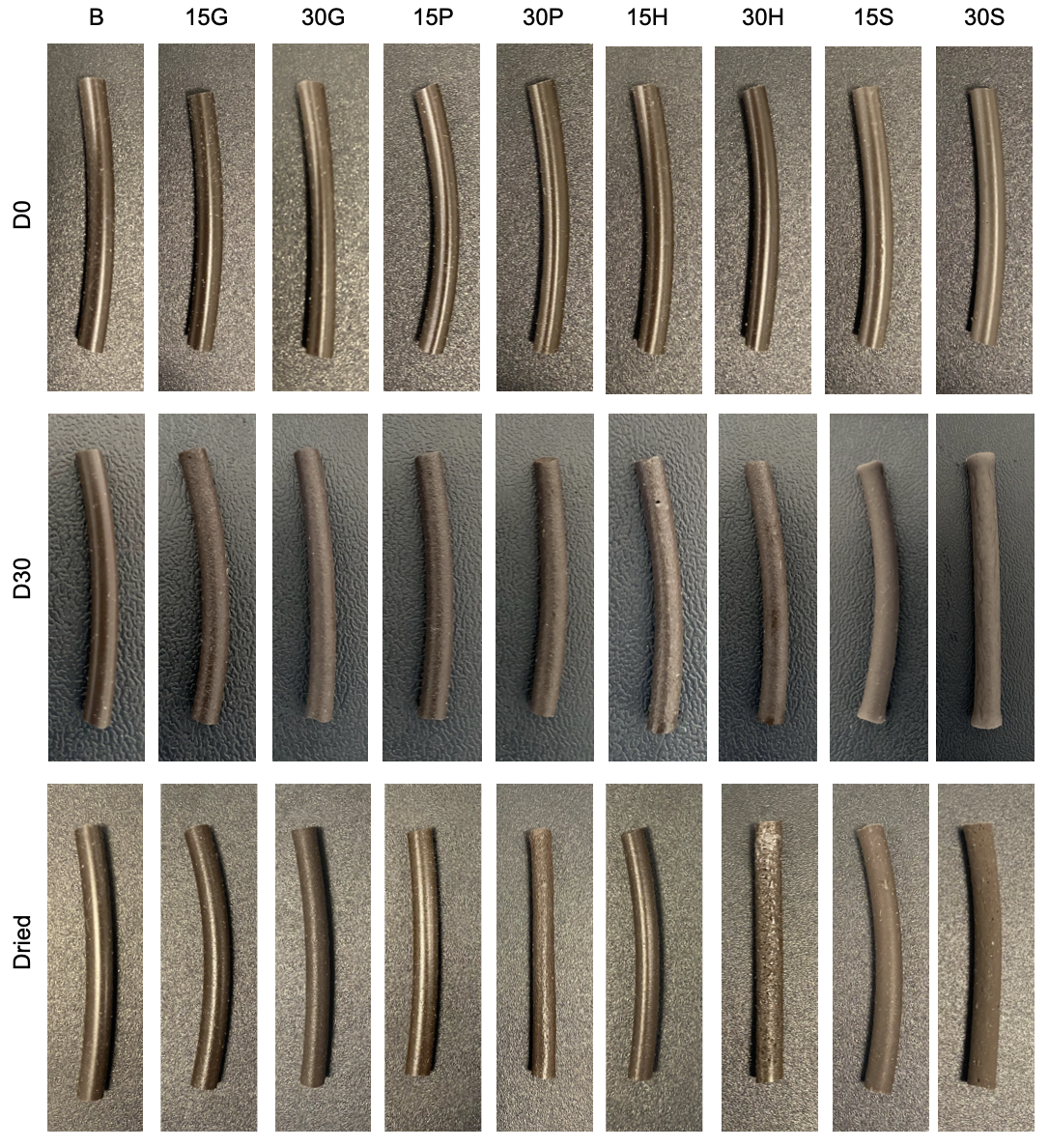


**Figure S1.** Representative photographs of formulations containing 5% w/w CN and 0, 15% w/w or 30% w/w gelatin, PVP, HPMC or sucrose before (D0), after in vitro release test (D30), and after drying post-release test (Dried). B represents formulation without excipient.


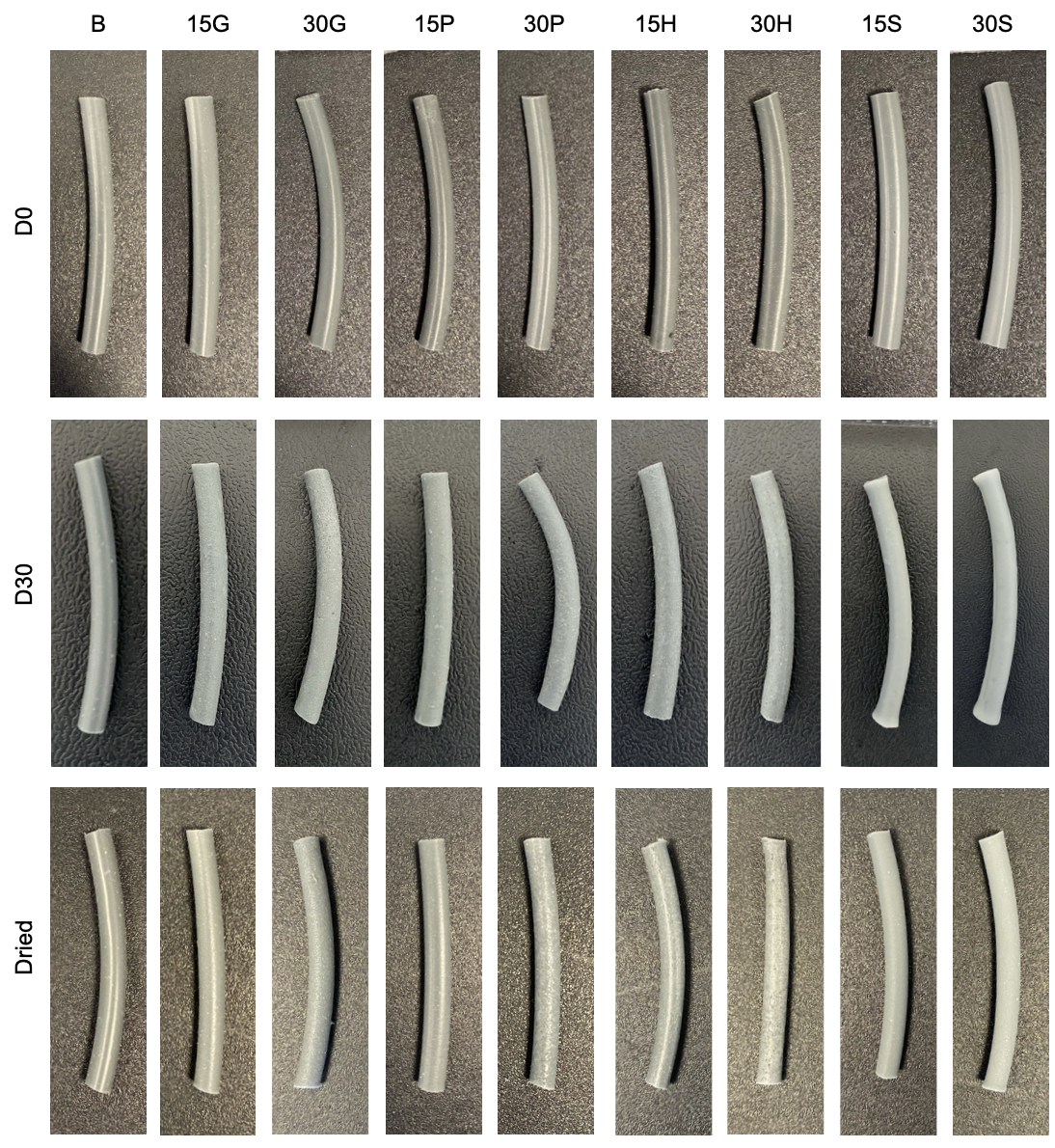


**Figure S2.** Representative photographs of formulations that contain 5% w/w ZN and 0, 15% w/w or 30% w/w gelatin, PVP, HPMC or sucrose before (D0), after in vitro release test (D30), and after drying post-release test (Dried). B represents formulation without excipient.


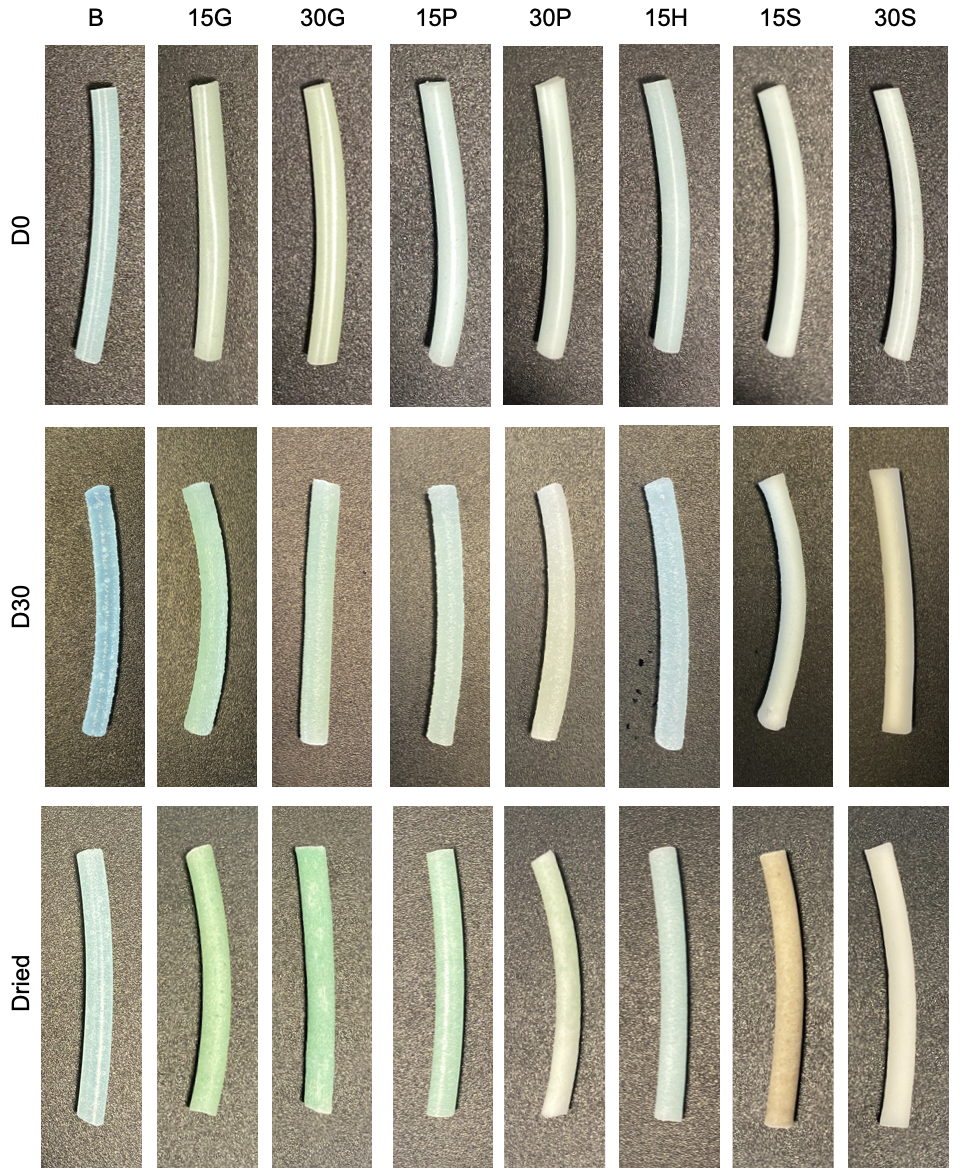


**Figure S3.** Representative photographs of formulations that contain 5% w/w CSP and 0, 15% w/w or 30% w/w gelatin, PVP, HPMC or sucrose before (D0), after in vitro release test (D30), and after drying post-release test (Dried). B represents formulation without excipient.


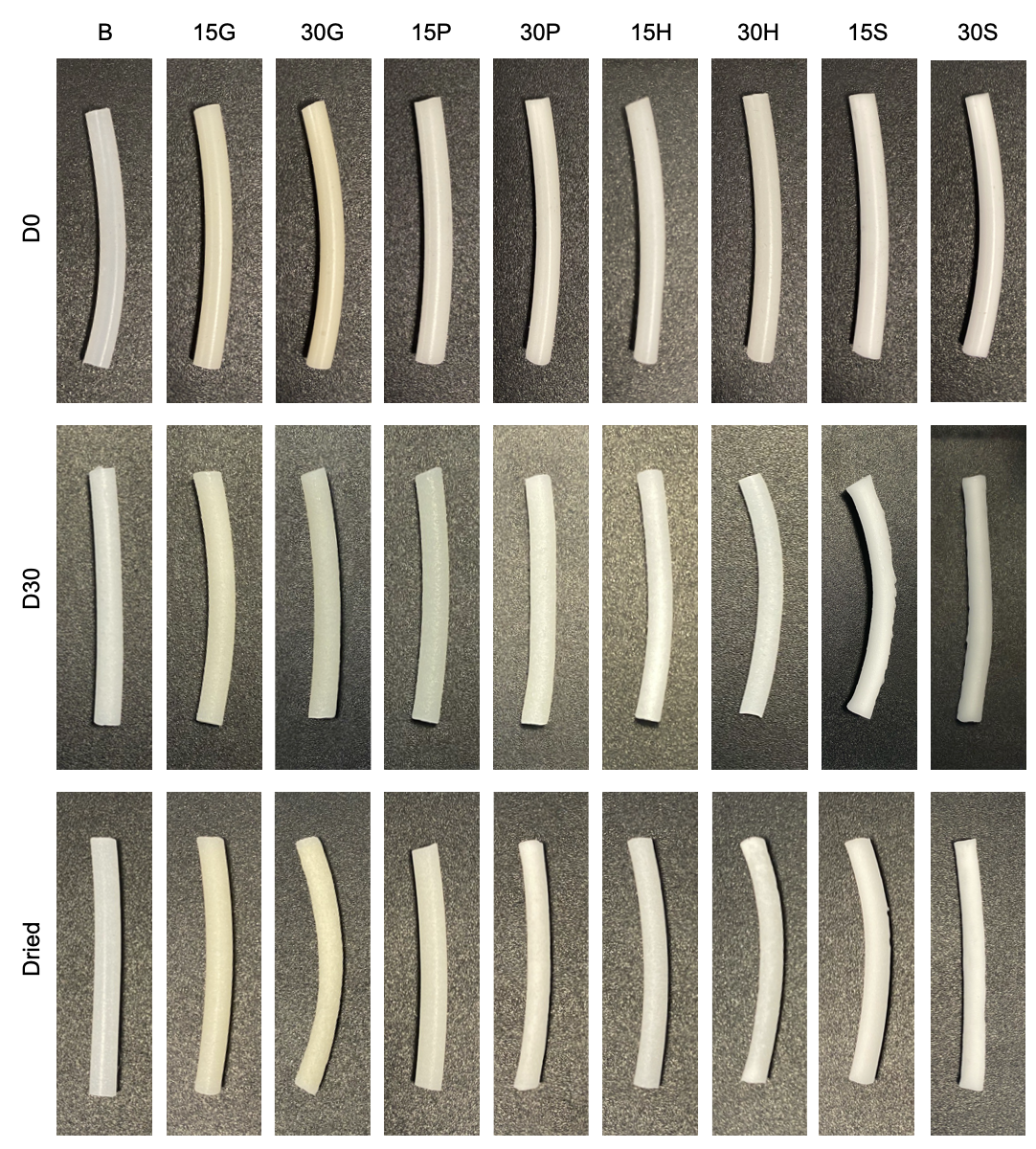


**Figure S4.** Representative photographs of formulations that contain 5% w/w ZAD and 0, 15% w/w or 30% w/w gelatin, PVP, HPMC or sucrose before (D0), after in vitro release test (D30), and after drying post-release test (Dried). B represents formulation without excipient.

**Figure S5.** Graphs showing cumulative Cu^2+^/Zn^2+^ release versus time (days) for matrix-type rods containing (A) CN, (B) ZN, (C) CSP and (D) ZAD.


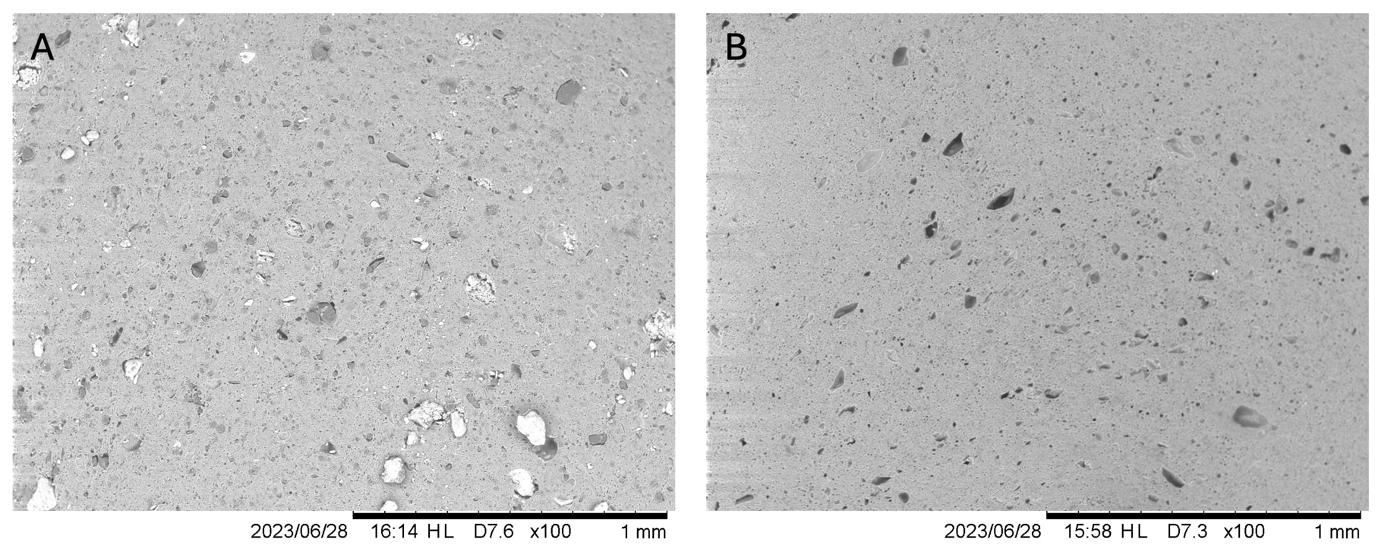


**Figure S6.** Scanning electron microscopy images for the cross-section of CSP-30S (A) after manufacture and (B) after drying post-release testing. The image (A) after manufacture shows the grey and white particulates corresponding to crystalline CSP and sucrose are clearly distributed across the rod matrix, while the image (B) after drying post-release testing shows the CSP and sucrose particles are almost no longer visible, suggesting that they were dissolved and leached out during release. The disappearance of these components implies the formation of porous channels, enhancing water ingress and facilitating metal ion diffusion.
